# AI-Driven Control Strategies for Biomimetic Robotics: Trends, Challenges, and Future Directions

**DOI:** 10.3390/biomimetics10070460

**Published:** 2025-07-14

**Authors:** Hoejin Jung, Soyoon Park, Sunghoon Joe, Sangyoon Woo, Wonchil Choi, Wongyu Bae

**Affiliations:** Department of Electrical Engineering, Soongsil University, Seoul 06978, Republic of Korea; wjdghlwls1234@soongsil.ac.kr (H.J.); syee20221239@soongsil.ac.kr (S.P.); wint2rbird@soongsil.ac.kr (S.J.); wsy04018@soongsil.ac.kr (S.W.); dnjsclf1456@soongsil.ac.kr (W.C.)

**Keywords:** AI-driven control, AI-algorithm, biomimitic robot

## Abstract

Biomimetic robotics aims to replicate biological movement, perception, and cognition, drawing inspiration from nature to develop robots with enhanced adaptability, flexibility, and intelligence. The integration of artificial intelligence has significantly advanced the control mechanisms of biomimetic robots, enabling real-time learning, optimization, and adaptive decision-making. This review systematically examines AI-driven control strategies for biomimetic robots, categorizing recent advancements and methodologies. First, we review key aspects of biomimetic robotics, including locomotion, sensory perception, and cognitive learning inspired by biological systems. Next, we explore various AI techniques—such as machine learning, deep learning, and reinforcement learning—that enhance biomimetic robot control. Furthermore, we analyze existing AI-based control methods applied to different types of biomimetic robots, highlighting their effectiveness, algorithmic approaches, and performance compared to traditional control techniques. By synthesizing the latest research, this review provides a comprehensive overview of AI-driven biomimetic robot control and identifies key challenges and future research directions. Our findings offer valuable insights into the evolving role of AI in enhancing biomimetic robotics, paving the way for more intelligent, adaptive, and efficient robotic systems.

## 1. Introduction

Living organisms have acquired highly sophisticated structures and functions through a long evolutionary process and natural selection over hundreds of millions of years. Beyond simple mobility, organisms have developed advanced survival strategies that encompass environmental perception, body control, sensory integration, learning, and social interaction. These biological characteristics are providing new design directions in robotics, and efforts to technically reproduce them have led to the emergence of biomimetic robots. Biomimetic robots initially started at the level of simply replicating the morphological features of organisms, but recently they are evolving toward engineering the sensory structures, motion patterns, and cognitive mechanisms of living organisms as well [1,2].

When classified based on function, such biomimetic robots can be divided into locomotion imitation, which implements movement strategies of living organisms such as walking, swimming, and jumping; sensory imitation, which replicates sensory mechanisms such as vision, touch, and hearing; cognitive and learning imitation, which implements neural networks, learning mechanisms, and swarm intelligence; and structural and regenerative imitation, which mimics biological structural resilience such as self-healing and flexible structures. For example, robots that reproduce the fin movements of fish achieve highly efficient propulsion in aquatic environments [3], and robot cameras modeled after insect compound eyes have achieved a wide field of view and the ability to maintain focus, contributing to the development of vision sensor technologies [1]. Robots that imitate the arm movements of octopuses [4] or autonomous underwater robots that mimic the swimming of sharks [5] can also be explained under this functional classification.

In particular, biomimetic robots are increasingly being used not only as tools for mimicking biological organisms, but also as scientific experimental tools for analyzing biological behavior. Experiments using robots are more controllable than those using animals, have excellent repeatability and reproducibility, and are advantageous in the standardization of experimental design. By mimicking the sensory–response–learning processes of living organisms in controlled environments, it is possible to quantitatively analyze specific cognitive processes, social behaviors, and neural responses, which has great academic significance [6].

Meanwhile, to meet the demands of complex structures and high-level control requirements in biomimetic robots, Artificial Intelligence technologies are recently being integrated as core control mechanisms. Reinforcement learning (RL) effectively reproduces how organisms adapt to their environment through trial-and-error-based behavior optimization [7], and deep learning enables real-time control by integratively processing various sensor data. Furthermore, research based on gene regulatory networks (GRNs) and evolutionary algorithms (EAs) is also showing examples where even the principles of morphogenesis in biology are being reflected in robot design [7,8].

Based on this background, this paper aims to systematically analyze the integration patterns between the functional structure of biomimetic robots and AI-based control technologies. In particular, focusing on the classification of biomimetic functions, this paper organizes the structure, control strategies, and applied effects of artificial intelligence algorithms used in each functional group based on actual research cases. Through this, it aims to comprehensively examine the interrelationship among biomimetic function–AI algorithm–control method, and to propose technical foundations and research directions for the future intelligence, autonomy, and application expansion of biomimetic robots.

**Figure 1:**
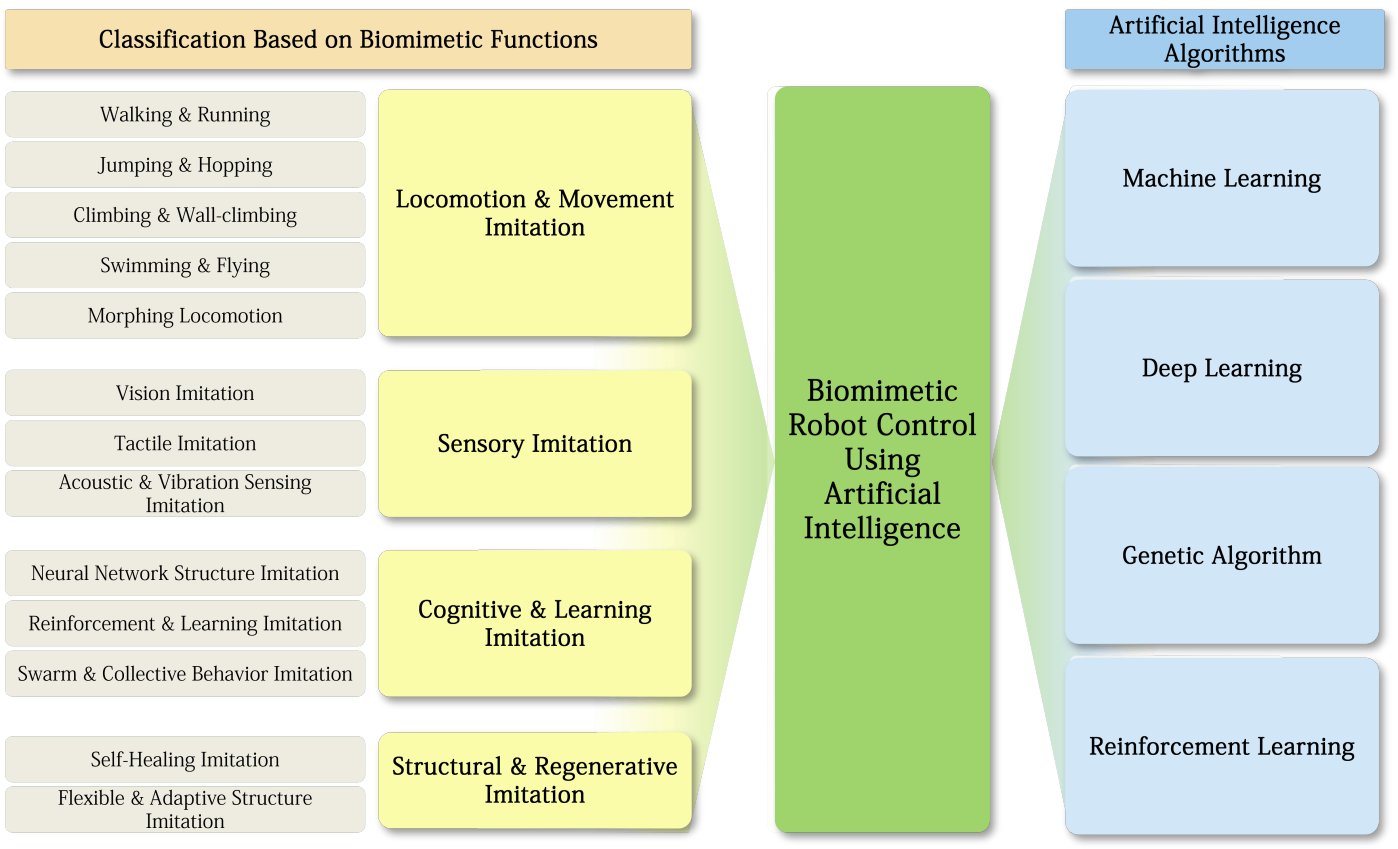
Biomimetic Robot Control Using Artificial Intelligence.

This review paper aims to systematically summarize the latest trends in biomimetic robot control technologies from the perspective of AI integration. In particular, based on the four functional classifications of biomimetics, it provides an integrated analysis of the structures of artificial intelligence algorithms and control strategies that have been actually applied in each functional group, and examines the interrelationship among biomimetic function–AI algorithm–control method. The frame of this article can be examined in the Figure 1 presented above.Through this, readers will be able to gain a multidimensional understanding of the evolutionary direction of biomimetic robot control technologies and the new possibilities offered by AI technologies.

## 2. Biomimetics

Biomimetic robots that imitate the behavioral mechanisms of animals evolved in nature have attracted great attention because they can implement flexible structures, high adaptability, and efficient motion patterns that conventional robots do not possess. These biomimetic robots originate from attempts to transfer the sensory, motor, and cognitive mechanisms of organisms such as animals, insects, and fish into artificial devices. In other words, biomimetic robots do not simply mimic structures, but aim to engineer the behavioral principles and neural-based control mechanisms of animals, thereby achieving much more robust and adaptive behavior compared to conventional systems. In this paper, biomimetic robots are classified based on functionality. When multiple functions are organically connected, classification is made according to the core mimetic function.

### 2.1. Locomotion Imitation

Locomotion imitation refers to the engineering reproduction of mechanisms by which organisms achieve efficient and flexible mobility in natural environments. This area is attracting attention for its potential to provide high adaptability and maneuverability even in complex terrains and environments that are difficult for conventional robots to handle.

#### 2.1.1. Walking and Running Imitation

Among biomimetic robots, those that implement walking and running motions mimic the locomotion mechanisms of humans or multi-legged animals. In particular, the design of leg movements in these robots is based on the skeletal structure, muscle arrangement, and gait patterns of biological organisms. Leg control in the hexapod walking robot Katherina was realized through a morphogenetic evolvable hardware (EHW) approach. A second case—a three-degree-of-freedom (3-DOF) bipedal robot leg—was designed to emulate human joint structures and muscle actuation. Both studies applied AI to joint-angle control, deliberately shaping each limb’s motion to closely mirror the kinematics of its biological counterpart [2,9]. In addition, in a wheel-type 2-DOF mobile robot that performs walking-like movements, a structure combining a bio-inspired backstepping controller that simulates the excitation and inhibition responses of the biological nervous system and a UKF-based state estimator was proposed. This system mathematically implements neuronal responses by simplifying the neuron membrane equations of the Hodgkin–Huxley model, and enables smooth and stable speed control even in real environments with sensor noise [10]. In this way, robots that imitate walking and running motions precisely reproduce the joint structure and sensory mechanisms of humans and animals, thereby ensuring real-time environmental responsiveness and walking stability. In particular, cases such as the EHW-based multi-legged robot and the 3-DOF bipedal robot demonstrate that the fusion of biological structural imitation and control algorithms can maximize the adaptability and flexibility of mechanical systems.

#### 2.1.2. Jumping and Landing Imitation

**Figure 2:**
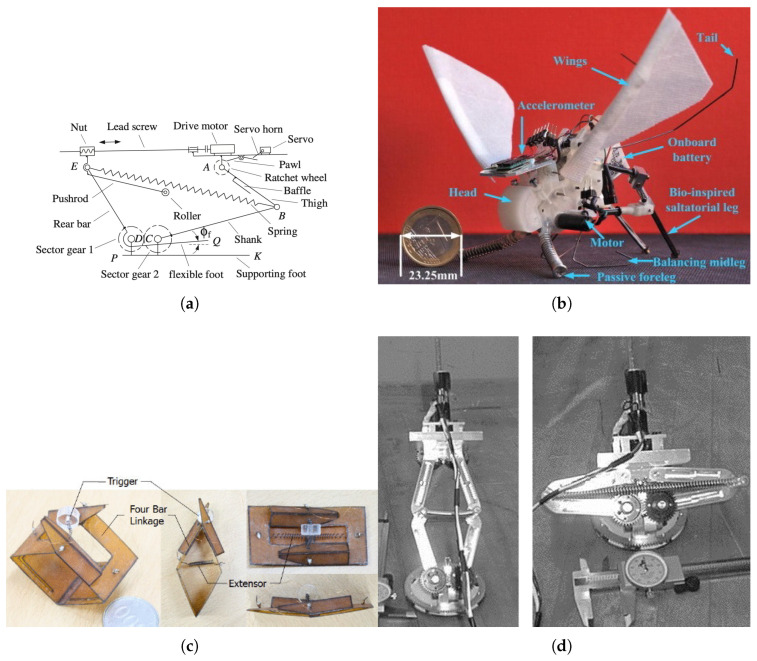
Structures of biomimetic robots for jumping and leaping: This is an example of focusing on reproducing the physical shape of each robot, a biomimetic robot using springs, catapults, and gears. (**a**) Autonomous jumping robot (adapted from [11]). (**b**) Grasshopper-inspired robot (adapted from [12]). (**c**) Flea-inspired robot (adapted from [15]). (**d**) Small jumping robot mimicking insects (adapted from [16]).

The jumping and leaping motions of animals are achieved by rapidly releasing a large amount of energy in a short period of time to generate high acceleration. The structural design of biomimetic robots for jumping and leaping can be seen in Figure 2. Small animals such as insects effectively perform jumping using a catapult mechanism that suddenly releases energy slowly accumulated in their muscles [11,12]. The mechanical implementation of the mechanism can be confirmed through Figure 2a. Moreover, cushioning strategies that minimize the impact when landing after a jump are also emerging.

Froghoppers exhibit particularly outstanding performance in such jumping mechanisms. Froghoppers use powerful muscles located in their hind legs and a unique locking mechanism to release forces up to about 414 times their body weight and leap more than 100 times their body length. The legs of the froghopper store elastic energy through muscle contraction just before jumping, and suddenly release this force through the unlocking of the latch mechanism [13].

A mini jumping robot shown in Figure 2b developed by mimicking these biological principles was designed by setting the tibia/femur ratio of the froghopper leg to 1.7 to implement optimized mechanical properties. This robot replaced biological muscles and tendons with a spring–gear mechanism, realizing high jump performance and efficient energy conversion. The spring–gear mechanism connects a spring to a gear responsible for rotating the first segment of the saltatorial leg, and by using a special gear for one-way rotation, effectively implements the jumping motion with a single-direction rotation. This robot can travel up to about twice its body height and four times its length in a single jump, while minimizing contact force and slippage risk during takeoff [12].

Robots that mimic the jumping method of insects like grasshoppers have also been developed. By analyzing the jumping mechanism of grasshoppers, a robot using a multi-link bar structure and logarithmic spiral gear was designed to achieve both mechanical simplification and efficiency. This robot ensures stable jump trajectories and minimal structural stress, showing kinematic characteristics similar to actual insects [14].

There is also a robot inspired by fleas. Based on the elastic energy storage and rapid release mechanism of fleas, a robot which shown in Figure 2c by using a catapult structure obtains high thrust during jumps. This robot performs effective jumping with a relatively simple mechanism and ensures stable mobility in various environments [15].

In research on jumping technologies for small robots in Figure 2d, robots that consider low-gravity environments have been developed. These robots are equipped with self-recovery systems and angle adjustment functions for post-jump attitude control and landing stability. By doing so, they recharge energy between jumps and perform jumping from a stationary state, enabling effective movement in complex environments [16].

Robots that mimic jumping, leaping, and landing motions realize high propulsion and efficient energy conversion by mimicking the elastic energy storage structures of insect legs in various ways. In particular, the jumping mechanisms of small insects are implemented with simplified elastic structures, leading to the development of jumping robots applicable to various sizes and environments. In the future, with the integration of technologies such as low-gravity adaptation, self-recovery functions, and enhanced landing stability, it is expected that the practicality and versatility of jumping-based locomotion systems will be further expanded.

#### 2.1.3. Climbing and Wall-Climbing Imitation

Robots that mimic the characteristics and structures of organisms capable of climbing vertically inclined surfaces such as walls are being developed in various forms depending on the method of surface contact. This section focuses on examples of robots inspired by the climbing mechanisms of animals such as geckos and rats.

Robots inspired by the remarkable climbing abilities of geckos have gained significant attention. Geckos possess exceptional adhesion capabilities due to their footpads densely covered with microscopic keratin fibers, allowing them to attach to walls regardless of surface roughness. Among two such robots developed to mimic this, the first one, the Rigid Gecko Robot (RGR), implements climbing using solenoid motors and adhesive pads made of highly adhesive Silly Putty. This robot simplifies the movement of gecko feet and is designed to allow the attached foot to adhere and detach easily by tilting it at a specific angle [17]. The second robot, the Compliant Gecko Robot (CGR), features a flexible spine structure using Shape Memory Alloy (SMA) wires and is fabricated at a smaller scale to increase usability in fine environments. CGR enables stable and energy-efficient climbing through the contraction and relaxation of the SMA wires [17]. Stickybot III mimics the flexible and hierarchical adhesive structures of gecko feet. This robot utilizes a gecko-like synthetic adhesive composed of synthetic microfibers and is equipped with a two-axis compliant mechanism that provides flexibility in the roll and yaw directions of the foot, allowing it to maintain uniform contact pressure even when alignment with the contact surface is imperfect. In addition, a tendon structure located at the rear of the foot uniformly distributes force to prevent detachment from the surface, thereby enabling stable climbing [18].

There is also a climbing robot developed based on studies of the inclined-surface walking characteristics of rats. Rats adjust the structure of their feet for efficient locomotion across various inclines and possess uniquely shaped footpads that minimize slipping in the direction of gravity. The robot mimicking this structure integrates flexible pads and sharp claws, enabling stable movement even on surfaces with varying slopes. This complex foot structure can respond to a wide range of surfaces, making it highly applicable in practical environments [19].

A robot utilizing a combination of flexible pads and claws has also been developed. This robot achieves strong adhesion and stable climbing on both smooth and rough surfaces through the composite structure of its claws and flexible pads. In particular, the robot’s claws effectively grasp and anchor onto the microstructures of surfaces, while the flexible pads provide additional friction and adhesive force to prevent slippage. This type of structure significantly improves the stability and reliability of robots in actual climbing environments [20].

In summary, climbing and wall-climbing robots are being developed through an integrated imitation of various structural and functional features of biological organisms, such as the microfiber-based adhesive mechanism and flexible joint structure of geckos, and the inclined-surface locomotion traits of rats. These robots are designed to adaptively attach to and move across surfaces depending on the type and gradient of contact, and their potential applications in both indoor and outdoor environments are continuously expanding.

#### 2.1.4. Swimming and Flying Imitation in Fluid Environments

Locomotion in fluid environments is a highly efficient mode of movement that many organisms in nature have developed through evolution, typically including swimming in water and flying through air. Recent studies have actively attempted to mechanically replicate the propulsion efficiency of biological organisms derived from organically controlled morphology, by observing and mimicking the motion principles of fish, marine animals, and birds. This section explores biomimetic approaches to locomotion in fluids through recent examples of developed robots. Studies on robots that mimic bird flapping flight have focused on how to enhance lift and use energy efficiently through wing shape and movement. Researchers fabricated robot wings inspired by bird flight and identified the aerodynamic mechanisms through which wing folding and stroke tilting improve flight efficiency. In particular, they confirmed that folding motions reduce turbulence and allow for more efficient propulsion [21].

Birds unfold or fold their wings and adjust the tail surface area depending on the environment to maximize flight performance. In this study, the aim was to improve the agility, energy efficiency, and stability of drones by engineering synergistic morphing. The wings and tail consist of nine artificial feathers each. The wings can increase their surface area by up to 41% and are designed to allow independent asymmetric deformation for roll control. The tail can increase its surface area by up to 214%, functioning simultaneously as an elevator and rudder. In attack flight, the wings are positioned forward and the tail is fully expanded, whereas in cruise flight, the wings are retracted backward and the tail is folded. This enables active control of wing and tail geometry during flight [22].

The development of robots that mimic aquatic organisms is also actively progressing. A representative example is a biomimetic fish robot equipped with multi-joint flexible fins. Inspired by the way real fish generate efficient propulsion through undulating fin motions, the fins were designed with a multi-joint structure to optimize propulsion, effectively reproducing the movements of biological fins [3].

Soft robotic fish using flexible materials have drawn attention for their enhanced underwater agility and environmental adaptability. The soft electronic fish proposed in this study adopts a motor-less actuation mechanism based on dielectric elastomers and ion-conductive hydrogels, achieving fast and flexible movement similar to real fish. The system integrates a silicone-based flexible body and fins, conductive hydrogel electrodes, and a compact electronic system with a high-voltage driving circuit. By using water itself as the grounding medium in the underwater environment, it resolves electrical insulation issues through an innovative design [23].

Research has also been conducted on an underwater hexapod robot with six legs. This robot implements insect-like walking motion in aquatic environments to generate propulsion, and its legs are designed to ensure stable forward movement underwater. It was confirmed that each leg’s movement provides both effective propulsion and stability in water [24].

He study by Behrens and Ruder applied biomimetic design to the field of microrobotics, developing a smart magnetic microrobot. This robot is only a few millimeters in diameter and is made from a soft magnetic polymer composed of agar hydrogel with uniformly dispersed iron oxide nanoparticles. The hydrogel is melted and injected into a 3D-printed helical mold, where it solidifies into a spiral structure. When a rotating magnetic field is applied, the robot spins and advances by scraping through the fluid in a helical motion. This helical propulsion mechanism is highly efficient in viscous fluids at low Reynolds numbers and resembles the way bacteria use rotating flagella for propulsion. The researchers chose this spiral soft robot design because it is a widely used motif in microrobotics for long-range control and enables effective movement with relatively simple structure under rotating magnetic fields [25].

A recent survey on the overall development of underwater robots analyzed the Body/Caudal Fin(BCF) propulsion method and the Median/Paired Fin(MPF) propulsion method, and compared how well robotic fish mimic the movement characteristics of real fish. The study revealed that biomimetic robots employing various propulsion methods exhibit low noise, high efficiency, and excellent maneuverability, highlighting their strong potential in underwater exploration and resource utilization [26].

In summary, robots that imitate locomotion in fluid environments precisely replicate the movement mechanisms of birds, fish, and aquatic insects, thereby achieving high propulsion efficiency, excellent agility, and adaptability. In particular, technologies such as morphing structures, flexible materials, and multi-joint mechanisms are being integrated to realize movements that closely resemble real organisms in both air and underwater environments. This supports the expansion of practical applications in search and rescue, marine exploration, and drone-based transportation in the future.

#### 2.1.5. Morphing Locomotion

Morphing locomotion refers to the mode of movement in which organisms change their shape using soft-bodied structures without bones, segmented body parts, or flexible joints—commonly observed in beetle larvae, earthworms, octopuses, and other species. The locomotion characteristics of such organisms provide advantages in maneuverability in confined spaces, flexible environmental adaptability, and self-reconfiguration capabilities, offering a new paradigm for robot system structural design and control methods. Recent studies have aimed to replicate the mechanisms of morphing motion, enabling robots to autonomously adjust their body shape and change movement patterns. This section introduces representative cases of morphing locomotion, including modular self-reconfigurable robots and continuum robots inspired by soft arms. In systems where multiple robot modules autonomously connect and operate as a single organism, the principle of morphogenesis in biology was mimicked to implement the ability to change body structures according to the environment. The study referenced the biological mechanism in which organisms regulate tissue development and structural formation using hormones, designing a system in which virtual hormonal signals are exchanged between robot modules to form the overall structure. This approach goes beyond simple structural connection, allowing the robot to adapt to diverse environments through autonomous structural formation and reconfiguration, and even evolve new movements by altering its structure when necessary. It was experimentally demonstrated that such organism-like robots can self-organize their form and evolve corresponding movement strategies in response to external environments [27].

Another notable study involves the STIFF-FLOP continuum robot, which mimics the flexible movement of soft-bodied animals like octopus arms. Octopus arms have a jointless, flexible continuum structure, yet are capable of highly dexterous and complex reaching motions. The STIFF-FLOP robot was designed to imitate these movements with the goal of realizing high-degree-of-freedom continuum reachability. The study collected motion capture data of actual octopus reaching behavior and parameterized it along three axes curvature, elongation, and rotation before training a probabilistic dynamical model. As a result, the robot successfully reproduced movements similar to those of real octopus arms, such as flexible extension through narrow passages and S-curve reaching motions without using joints. This biomimetic approach enables motion that passes through narrow spaces and extends flexibly in various directions, even without complex joint mechanisms [4].

These studies represent efforts to overcome the limitations of fixed mechanical structures and realize self-changing architectures and nonlinear, multi-degree-of-freedom motion control based on environmental feedback. Such approaches are expected to contribute substantially to applications in soft robotics, surgical robotics, and exploration robotics operating in complex environments. Future research is anticipated to further integrate these biomimetic structure-morphing technologies with advanced control algorithms and real-time learning mechanisms, ultimately leading to the development of true bioinspired robots capable of simultaneous morphological and functional adaptation.

### 2.2. Sensory Imitation

#### 2.2.1. Vision Imitation

Vision imitation refers to the technology that mimics the visual structures and optical principles of biological organisms and applies them to robots or electronic devices. In nature, various organisms have evolved exceptional visual abilities in response to their environments, and studies aiming to engineer these biological mechanisms have been actively conducted. In particular, the compound eyes of insects have drawn significant attention in recent biomimetic vision research due to their wide field of view, excellent motion detection capability, and infinite depth of field.

**Figure 3:**
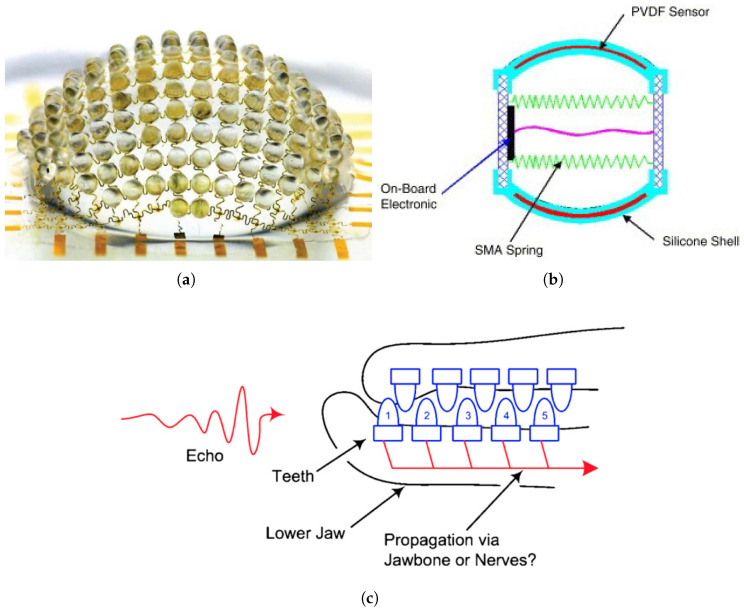
Sensory imitation: It shows an example of actual reproduction of visual, tactile, and auditory and vibrational sensory imitations. It can be seen that the focus was on biomimetic reproduction of hardware rather than signal control. (**a**) Mimicry of arthropod compound eyes (adapted from [1]). (**b**) Mimicry of earthworm tactile mechanism using PVDF film(adapted from [28]). (**c**) Mimicry of dolphin vibration sensing (adapted from [31]).

Song et al. [1] developed a hemispherical digital camera that mimics the compound eyes of arthropods Figure 3a, overcoming the limitations of conventional planar sensor technologies. This camera was inspired by the apposition compound eye observed in insect vision and was constructed in a structure where individual microlenses correspond to each photodiode to form artificial ommatidia. An innovative fabrication process was applied, in which the optical component made of polydimethylsiloxane (PDMS) based elastomer and the electrical component composed of a thin silicon photodiode array were elastically deformed from a flat state into a hemispherical shape [1].

This biomimetic camera features a wide field of view of approximately 160 degrees. The curvature and arrangement of the microlenses were optimized to prevent visual overlap between adjacent lenses. Additionally, the elastic mechanical response characteristics were designed to maintain optical performance even under large deformation. The researchers numerically and experimentally confirmed that the mechanical strain occurring during the elastic deformation process does not affect the overall optical performance of the array [1].

The advantage of this hemispherical visual structure is that it can simultaneously secure a wide field of view and infinite depth of field, which are difficult to achieve with conventional flat sensors and lens systems. In fact, the developed biomimetic camera captures clear images across a very wide visual range similar to that of an insect compound eye. As the object distance increases, the image size becomes smaller, but the focus is maintained without blurring, realizing the infinite depth of field characteristic [1].

Such research demonstrates that the advantages of biological visual structures can be applied to digital camera technologies, contributing to the development of robots and electronic devices with more advanced visual performance. In the future, it is expected that the ability to more effectively mimic the complex and sophisticated vision systems found in nature will be enhanced through miniaturization and diversification of array configurations.

#### 2.2.2. Tactile Imitation

Tactile imitation technology refers to systems designed to mimic the skin or tactile organs of biological organisms, enabling robots to accurately detect interactions with the external environment as well as internal state changes. Recent research has focused on analyzing the tactile mechanisms of various organisms to develop sensors with high sensitivity and flexibility.

A tactile sensor inspired by the tactile mechanism and proprioception (muscle stretch detection) of earthworms was implemented using a flexible polyvinylidene fluoride(PVDF) film Figure 3b. PVDF exhibits high sensitivity and excellent flexibility, and it is particularly capable of detecting both external contact stimuli (exteroception) and proprioception. The research team fabricated the PVDF film in a perforated form and embedded it into a flexible silicone structure, thereby developing a sensor that allows the robot to sensitively detect both contact with the surrounding environment and deformation of its own body [28].

Another example is the development of hydrogel-based electronic skin, which realizes tactile functions similar to actual biological skin. This sensor utilizes the outstanding flexibility and biocompatibility of hydrogel and is applied to the surface of robotic skin. Tactile information is obtained by measuring changes in electrical signals due to mechanical deformation. In particular, its high stretchability allows it to be used in various fields such as soft robots and wearable devices, where large deformations occur with movement [29].

There is also a tactile sensor developed for underwater robots with soft bodies, such as the lamprey. This study was inspired by the skin-sensing mechanism of lampreys, which possess soft, flexible bodies, and designed an extremely flexible and resilient sensor system. This sensor quickly detects subtle pressure changes in fluid environments, enabling the robot to precisely perceive its surroundings. The researchers embedded the highly flexible sensor into a lamprey-inspired robot and demonstrated that the robot could respond immediately to changes in its environment [30].

Tactile imitation technology continues to advance by mimicking the sensory mechanisms of various biological systems such as earthworms, lampreys, and natural skin, enabling robots to detect both external stimuli and internal conditions simultaneously. PVDF film-based sensors can recognize external contact and body deformation concurrently, while hydrogel electronic skin offers tactile capabilities suitable for soft robots due to its high stretchability and biocompatibility. Furthermore, the development of flexible sensors capable of precise detection in aquatic environments is greatly enhancing robotic interaction capabilities in complex environments.

#### 2.2.3. Acoustic and Vibration Sensing Imitation

Acoustic and vibration sensing imitation refers to technologies that mimic how animals in nature detect their surroundings through sound or vibrations, implementing such mechanisms as sensory systems for robots. In particular, animals such as dolphins and bats possess highly developed auditory systems known as echolocation, and recent research actively seeks to replicate these capabilities.

Dolphins demonstrate excellent detection performance even in shallow and turbid marine environments, significantly outperforming conventional artificial sonar systems. Among the auditory mechanisms of dolphins, recent studies have focused on the lower jaw, which functions as a major receiving component in the echolocation system Figure 3c. Dolphins possess a series of teeth arranged at regular intervals in the lower jaw, and it has been hypothesized that this tooth arrangement acts as an endfire array for acoustic reception. In this system, each tooth serves as a resonant pressure sensor for receiving sound waves, and the acoustic signals are transmitted along the jawbone to the inner ear [31].

The researchers confirmed that the tooth arrangement in dolphins has directionality, selectively amplifying sound waves approaching from the front. This array structure combines signals received at each tooth with different time delays, reinforcing only the signals arriving from specific directions and thereby offering very high angular resolution. In particular, a structure known as the monopulse method—where two arrays are placed at slightly different angles—allows the system to determine the direction of the signal precisely using only a single pulse [31].

This endfire array structure maintains high directionality even at short distances, which makes it highly suitable for applications such as underwater exploration and mine detection, where precise detection of targets at close range is essential. Additionally, the research team analyzed the dolphin’s tooth arrangement as a log-periodic array, a configuration that maintains stable directionality across a wide range of frequencies. This model has been proposed to explain how dolphins effectively receive acoustic signals under various soundscape conditions [31].

Acoustic and vibration sensing imitation technology is progressing toward the development of biomimetic reception systems based on the echolocation mechanisms of dolphins, offering high-resolution directionality and the ability to operate across wide frequency ranges. In particular, endfire and log-periodic array structures that mimic the tooth arrangement of the dolphin’s lower jaw maximize the directionality and angular resolution of acoustic signals. Through the application of the monopulse method, these systems can accurately detect directions with a single acoustic pulse. Such technologies are gaining attention as next-generation acoustic perception platforms that can supplement or replace conventional sonar systems in underwater exploration, close-range acoustic sensing, and target detection in low-visibility environments.

#### 2.2.4. Proprioceptive Sensing Imitation

Proprioception is an internal sensory modality that underpins motor control by continuously monitoring the position, posture, equilibrium, and force, in real time, within muscles, joints, and tendons. In living organisms, proprioceptors—such as muscle spindles and Golgi tendon organs—relay signals incessantly to the brain and spinal cord, enabling precise body-state awareness even in the absence of exteroceptive cues.

Recently, robotics research has focused on mimicking biological proprioception by developing various internal-state sensing technologies, including flexible and stretchable sensors, soft sensors, and fiber-optic-based sensors. Traditional robotic systems have primarily relied on sensing external environmental information through vision sensors and tactile sensors. However, such exteroceptive sensing approaches exhibit limitations due to vulnerability to visual occlusion, illumination variations, environmental noise, and external disturbances, making it challenging to promptly and accurately determine the robot’s internal state. In contrast, proprioception enables rapid and reliable measurements of internal states, such as joint positions and structural deformations, independent of external environmental conditions, thus facilitating robust control and self-state estimation even in unexpected scenarios.

Such research has been particularly active in the fields of soft robotics inspired by cephalopods such as octopuses, as well as articulated robotic systems like robotic arms and legs. Recently, integrated control architectures combining proprioceptive and exteroceptive sensing have been proposed, leveraging the complementary nature of these sensory modalities. This hybrid approach has demonstrated effectiveness in overcoming inherent weaknesses such as sensor failure and environmental uncertainties.

Cianchetti et al. [32] proposed a biomimetic proprioceptive sensing architecture inspired by the octopus arm, integrating distributed stretch sensors and contact sensors into an OCTOPUS robot equipped with eight soft arms. Experimental results demonstrated that signals from the embedded stretch sensors allowed real-time reconstruction of the robot’s joint angles and deformation states. Specifically, joint angles of arm segments were estimated with an average error of less than 6 degrees, and the embedded contact sensors achieved a detection success rate exceeding 85% across various gripping tasks. Utilizing this internal-state feedback, the OCTOPUS robot successfully performed diverse shape manipulations and adaptive movements without relying on external visual input. These results highlight that comprehensive proprioceptive feedback beyond simple local sensing can significantly enhance robotic adaptability and control performance in complex environments [32].

Wensing et al. [33] applied proprioception-based actuator design to the MIT Cheetah 2 quadruped robot. This study integrated high-resolution encoders and torque sensors within each leg joint, enabling the robot to precisely monitor internal states such as joint position, velocity, and torque in real-time. The proprioceptive sensing architecture significantly contributed to the robot’s capability to respond instantaneously to dynamic events such as collisions, slippage, and contacts with external environments, thereby achieving robust balance maintenance and adaptive locomotion. Furthermore, force and impedance control strategies leveraging proprioceptive feedback notably improved stability and energy efficiency of the robot under external disturbances [33].

Lloyd et al. [34] proposed a non-prehensile manipulation approach by combining high-resolution optical tactile sensing with proprioceptive feedback from a robotic arm. This approach integrates local contact information obtained from the tactile sensor and proprioceptive data from robot joints, demonstrating precise estimation and control of both object and end-effector positions without relying on external models or visual inputs. Experimental results validated the method’s robustness across diverse object shapes and initial positions on planar and curved surfaces [34].

Recently, proprioceptive learning methods have been proposed, such as the study by Liu et al., where an internal vision system within a soft robotic structure was combined with deep learning to precisely estimate real-time three-dimensional deformation, force, and torque of a finger-like structure [35].

Such biomimetic proprioception research significantly enhances the robot’s self-state estimation accuracy and adaptability, providing a robust foundation for achieving reliable control and real-time interactions even in complex and highly dynamic environments.

### 2.3. Cognitive and Intelligence Imitation

#### 2.3.1. Neural Network Imitation

Artificial systems that mimic the structure of biological neural networks have been extensively studied in terms of both efficient information processing and the reproduction of biological functions. This section explores studies that aimed to mimic neural network structures as a foundation for developing AI technologies and to more precisely replicate biological mechanisms.

Krichmar et al. [36] proposed a biomimetic strategy called Biomimetic Research for Energy-efficient AI Designs(BREAD), inspired by the structure of the human brain. This study highlighted how biological brains perform complex information processing despite extremely limited energy consumption. It suggested that the connectome (neural connectivity structure), spike-based sparse coding considering energy efficiency, and dimensionality reduction techniques that maximize the mutual information between input and output signals of the neural network could be applied to AI systems to achieve the information processing efficiency of biological neural networks [36].

Levi et al. [37] developed a biomimetic neural network through the BrainMorphic Artificial Intelligence(BMAI) project that precisely replicates the behavior of biological neurons. In this study, analog and digital silicon neurons were implemented, reflecting the actual characteristics of biological neurons. In particular, they accurately reproduced the diverse spike generation patterns observed in neural signal conduction, significantly enhancing biological fidelity compared to conventional leaky integrate-and-fire models [37]. As a result, the study enabled the implementation of neuromorphic systems that more realistically mimic the behavior of biological neurons.

There has also been a study aiming to reproduce the memory and cognitive characteristics of brain neural circuits using reverberating neural networks. This research proposed a neural architecture in which circulating neural signals realize short-term memory and continuous reinforcement of information, offering a way to replicate biological short-term memory functions within artificial systems [38].

Additionally, research has been conducted on the implementation of multicellular morphological robots that integrate morphogenetic development with motor control, utilizing morphogenetic evolution. This approach proposed a structure in which neural networks and robot morphology evolve interactively, enabling the robot to autonomously change its form and movement strategy according to the environment [7].

Studies that imitate biological neural network structures aim to develop more bio-inspired and efficient intelligent systems by integrating the core principles of biological systems—such as energy efficiency, signal transmission characteristics, and evolutionary adaptability—into AI design. BREAD and BMAI enhanced the precision and effectiveness of neuromorphic chip design by focusing on neural connectivity and spike-based processing. Meanwhile, reverberating neural networks and morphogenetic neural structures attempted to engineer higher-level brain functions such as memory, adaptation, and morphological formation. These studies illustrate the potential of biomimetic algorithms that differ from conventional deep learning, and are expected to drive the evolution of AI control systems toward greater autonomy and adaptability.

#### 2.3.2. Learning Imitation

The learning process of biological organisms is similar to Reinforcement Learning in that it finds optimized behaviors through trial and error in interaction with the external environment. In particular, RL mimics the concept that organisms evaluate and learn behaviors through rewards, and it is effectively utilized for optimizing autonomous robot behavior. Recently, as RL has been successfully applied to underwater biomimetic robot control, it has gained attention for solving complex motion optimization and decision-making problems that are difficult to address with conventional control techniques.

In the study by Schramm et al. [7], a method was proposed that imitates the co-evolution of biological neural systems and body morphology to simultaneously evolve the shape and behavioral control mechanisms of robots. The model used in this research employs a GRN to grow the robot’s morphology while concurrently developing its motion control strategies. As a result, the robot’s body form and control strategy are tightly integrated, enabling the formation of efficient locomotion strategies in its environment [7].

Reinforcement learning, which imitates the reward-based learning principles of living organisms, contributes significantly to enhancing autonomy, adaptability, and control capabilities in complex environments. Particularly in the case of biomimetic underwater robots, DRL enables the robot to learn effective control policies without relying on physical models, demonstrating stable performance even under uncertain and noisy conditions. Furthermore, the GRN-based approach for co-evolving body and control presents a new paradigm that organically combines morphological and behavioral strategies, offering an engineering realization of biological adaptation mechanisms.

#### 2.3.3. Swarm and Collective Behavior Imitation

**Figure 4:**
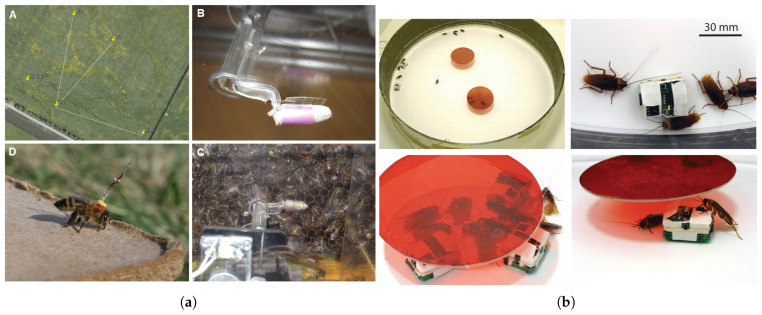
Examples of swarm behavior imitation methodology: (**a**) Honeybee tracking and marking using harmonic radar. A—Radar, Hive, feeder layout. B—Close-up of the RoboBee. C—RoboBee from the inside of the hive. D—Honeybees with transponders for radars (adapted from [39]). (**b**) Experimental arena for observing cockroach aggregation behavior. The pheromone robot shown in the top right was used. The bottom panels depict changes in aggregation behavior as a function of shelter illumination and robot placement. (adapted from [6]).

Swarm behavior refers to the emergence of complex collective actions at the group level through simple rules and interactions among individual agents, commonly observed in social insects such as ants, bees, and cockroaches. In biomimetic robotics research, efforts have been actively made to reproduce such swarm behaviors in order to implement autonomous and distributed cooperation among multiple robots. A robotic study that mimicked the swarm behavior of honeybees involved a robot called RoboBee, which replicated the waggle dance performed by bees to communicate the location of food. Real bees observed and responded to the dance of the robot shown in Figure 4a, successfully reproducing social communication mechanisms within the swarm. This demonstrated the robot’s ability to guide the flight paths of real bees, providing a key example of how robots can mimic and convey social signals to influence biological behavior [39].

In a study that imitated the social behavior of cockroach swarms, robots were integrated into a group of real cockroaches to observe their interactions. Figure 4b illustrates the behavioral tendencies of the cockroach swarm. In this setup, the robot communicated with the cockroaches using chemical signals and led them to make collective decisions such as selecting a specific shelter. Remarkably, despite being in the minority, the robots were able to influence the group’s final decisions, highlighting the importance of social feedback in decision-making within mixed natural–robotic swarm systems [6].

There have also been studies on the behavioral control of robot swarms themselves. Macroscopic modeling techniques, such as spatial continuum and compartment models, were used to predict and model the spatial distribution of biological swarms. Through this, the robots were able to autonomously gather in specific locations in response to environmental stimuli such as light. This study showed that swarm robots can form complex group behaviors through interactions with the environment based on simple individual rules [40]. Inspired by morphogenesis, the self-organizing process in embryonic development where cells autonomously position and organize themselves based on gene expression and morphogen concentration gradients, one study proposed a robotic swarm framework for collective pattern formation and boundary coverage. This system, based on an artificial GRN, allowed robot swarms to autonomously form and maintain patterns along environmental boundaries. The framework was designed to operate without requiring knowledge of the total number of robots or global coordinate information and enabled complex 2D and 3D shapes to be formed through only local interactions among robots. Simulations showed that the proposed method maintained high flexibility and stability even during shape formation, boundary coverage, obstacle avoidance, and the addition or removal of robots [41].

Similarly, an approach inspired by hierarchical GRNs enabled adaptive control of collective robot pattern formation in response to environmental changes. Using the GRN structure, robots operated in a distributed manner and autonomously adjusted swarm-wide pattern formation, achieving group behavior capable of actively adapting to changing conditions [42]. Swarm behavior imitation is gaining attention as a key strategy for implementing autonomous and distributed group intelligence by applying the simple rule-based interactions of social insects to robotic systems. Interaction with cockroaches and bees demonstrated that robots can intervene in or guide the decision-making processes of real organisms. Meanwhile, studies using macroscopic modeling and morphogenetic principles have successfully realized collective responses and autonomous pattern formation in robot swarms in response to environmental stimuli. In particular, GRN-based hierarchical control enables group behaviors with both environmental adaptability and scalability, significantly advancing the engineering feasibility of biological swarm intelligence.

### 2.4. Structure and Restoration Imitation

#### 2.4.1. Self-Healing Imitation

Biological organisms possess self-healing abilities that allow them to recover from damage, and research has sought to imitate these traits in engineering to enhance the durability and adaptability of robots. Two recently reported approaches implement self-healing mechanisms in distinct ways: one involves a robot composed entirely of living cells, known as a xenobot, which autonomously heals wounds through inherently programmed cellular behavior; the other utilizes a biohybrid muscle actuator, where actuation induces the strengthening of muscle tissue itself. In both cases, structure or performance is recovered and improved without external intervention, presenting robots that are robust to damage and capable of long-term stable operation.

Blackiston et al. [43] developed a small biological robot composed of embryonic cells from the frog Xenopus laevis, referred to as a xenobot, and observed its ability to self-repair following physical injury. Initially formed as a spheroidal multicellular tissue, xenobots demonstrated immediate self-healing responses when experimentally subjected to lacerations approximately halfway through the body using a scalpel. Surrounding cells migrated toward the wound, and the tissue edges contracted, resulting in complete closure of the wound within 15 min and restoration of the original spherical form. Notably, no xenobots died as a result of the injury. This rapid sealing is attributed to the innate regenerative capacity of amphibian embryonic tissue; similarly sized tadpole tissues are also known to self-repair quickly after mechanical damage. Inside the xenobot, individual cells cooperate to close wounds via cell–cell interactions, and new shapes are formed through self-organization, similar to embryonic development. The authors noted that for small injuries, active tissue contraction by the cells was sufficient for closure, although the mechanisms for repairing larger damage remain unclear. Xenobots restore injuries through the intrinsic healing instincts of the cells without any software or artificial structures. This capability represents a biological advantage that is difficult to replicate in synthetic-material-based robots [43].

Schaffer et al. [44] proposed a concept oftraining biohybrid robots by exercising them, applying the phenomenon of activity-induced strengthening in biological muscle tissue to robot design. In living organisms, skeletal muscles become thicker and stronger through the recovery process after repeated micro-damage, a phenomenon known as exercise-induced adaptation, which serves as an important biological self-healing mechanism. The researchers constructed a simulation model in which worm-shaped soft robot lattice structures were embedded with multiple artificial muscles and trained via reinforcement learning to reach target points. Crucially, they introduced a biomimetic adaptation algorithm in which the maximum contraction force of the muscle actuators increased depending on the prior activity history. At each episode, the maximum strain and stress experienced by the muscle contributed proportionally to an increase in the actuator’s force limit in the following episode, with a mathematical function ensuring a gradual increase. This mimicked the mechanotransduction pathway, where mechanical load triggers protein synthesis and cellular growth signals in muscle fibers. In actual tissue engineering experiments, repeated mechanical stimulation has been observed to enhance muscle contractility. In simulation, the adaptive muscle system led to spontaneous improvement in robot movement over time, showing higher rewards and faster convergence compared to non-adaptive systems under the same control algorithm. This was because the robot, initially starting with weak force, gradually gained greater actuation power through self-reinforcing muscles, thereby improving its ability to perform difficult tasks and increasing learning efficiency. In short, by mimicking exercise-induced self-healing adaptation in biology, the study confirmed the effect of improving long-term motion performance in robots [44].

#### 2.4.2. Flexible and Adaptive Structure Imitation

Flexible structure imitation refers to an approach that applies the deformable and adaptable physical structures of biological organisms to artificial systems. Many organisms in nature adapt to complex environments and move efficiently through soft bodies and variable shapes. By observing and mimicking these biological strategies, artificial robots can also learn or exhibit adaptive behaviors in unpredictable environments. Recent robotic research has increasingly adopted systems with composite materials and dynamic morphology, which improve functionality but also pose challenges for conventional modeling and control [25]. Accordingly, attention is shifting beyond rigid robotic structures toward soft-bodied robots and biomaterial-based robots, with growing interest in leveraging biomimetic advantages such as self-healing, shape transformation, and environmental adaptability. Ultimately, flexible structure imitation may lead to the development of novel mechanical systems with self-regeneration, biodegradability, and biocompatibility, which are difficult to achieve with conventional metal- or plastic-based technologies [45].

Kriegman et al. [45] proposed a method for designing fully biological robots that perform desired behaviors using contractile cardiac cells and non-contractile epidermal cells from frog embryos as building blocks. They evolved random cell combinations in a virtual space using an evolutionary algorithm to optimize the shape to perform a specific target behavior, such as maximizing the distance traveled, and quickly implemented the result into actual cell assembly. The resulting millimeter-scale living machines performed target functions with various unique morphologies, which did not resemble traditional organs or tissues found in nature but consisted of entirely new structural combinations. For example, evolved designs were expressed as patterns of contractile cardiac cells and structural epithelial cells arranged in a virtual lattice shape [45], and based on this, actual biological tissues were layered and shaped into similar shapes through micromanipulation to implement the designed shapes as real living tissues [46]. These reconfigurable organisms demonstrated basic behaviors such as autonomous movement and environmental exploration for several days or even weeks without additional nutrient supply [45] and some designs exhibited morphology-dependent behaviors, such as collective interaction or object-pushing locomotion.

Another notable case involves the design and optimization of a dynamic building façade system using Ethylene-Tetrafluoroethylene (ETFE) cushion units, inspired by phenotypic variability in biology—especially adaptive structural responses to environmental changes. In nature, phenotypes are the result of interactions between genotype and environment, allowing organisms to adapt to surrounding conditions. This study mimicked such biological principles by considering each cushion in the building facade as a “gene” and implemented a digital phenotypic system in which the entire system adaptively evolves in response to external environmental factors and internal user needs. The façade system consists of 100 ETFE cushions, each of which is designed to autonomously open or close depending on variations in temperature and solar radiation. The overall system learns and optimizes collective adaptive behavior [47].

## 3. Artificial Intelligence

Artificial Intelligence refers to technologies designed to enable computers to perform intelligent behaviors by mimicking human cognition, learning, reasoning, and problem-solving capabilities. Since its conceptual foundation in the 1950s, AI has undergone rapid advancement. Initially centered around logic-based rule systems, AI has since diversified into various approaches including machine learning based on empirical learning, deep learning utilizing deep neural networks, RL that acquires behavior based on reward through interaction with the environment, and EAs that mimic the principles of biological evolution. These methods now play a central role in the automation and optimization of complex systems [48].

AI is being widely applied across modern industry and science, including in autonomous driving, smart manufacturing, medical image analysis, natural language processing, and predictive systems. In particular, it has recently emerged as a core technology in the field of biomimetic robotics. While biomimetic robots reproduce the sensory and motor mechanisms of animals to achieve high adaptability and mobility, traditional model-based control approaches face limitations in enabling robots to autonomously make decisions and generate optimal behaviors according to environmental conditions. AI offers a critical turning point in this regard, as it enables robots to learn from data like living organisms and autonomously adjust behavior in response to changing external environments, thereby providing cognitive flexibility.

For example, methods that co-evolve robot morphology and control strategies using GRNs represent an application of biological development and adaptation mechanisms to robotics, demonstrating the potential for robots to optimize both body structure and motion in complex environments [7]. Furthermore, in GRN-based collective robot control studies, individual robots autonomously responded to environmental changes while collectively forming structural patterns at the swarm level [42]. This approach mimics the integrated characteristics of sensing, processing, and responding as seen in biological nervous systems, forming the basis for extending biomimetic robots beyond mere imitation into intelligent behavior generation systems.

Meanwhile, AI algorithms have become essential technologies that enable robots to make stable decisions and perform actions even in complex and uncertain environments. Reinforcement learning, in particular, enables policy learning through interaction with the environment without requiring explicit mathematical models, making it suitable for robust control in real-world conditions characterized by nonlinearity and disturbances. DRL has greatly expanded the scope of RL by allowing policies to be approximated and learned in high-dimensional and nonlinear environments. The initial Deep Q-Network(DQN) achieved human-level performance by interpreting Atari game screens via Convolution Neural Networks(CNNs) [49], followed by policy-based algorithms such as Trust Region Policy Optimization (TRPO) and Proximal Policy Optimization (PPO) that improved training stability [50,51]. Asynchronous Advantage Actor-Critic (A3C) enhanced sample efficiency through multithreaded parallel learning [52], and more recently, off-policy continuous control algorithms such as Soft Actor-Critic (SAC) and Twin Delayed Deep Deterministic Policy Gradient (TD3) have shown excellent performance in quadruped locomotion tasks [53,54]. Additionally, hybrid approaches that combine Spiking Neural Networks (SNNs) with DRL have demonstrated potential for improved energy efficiency and learning speed, paving the way for future research into robust, autonomous, and energy-efficient biomimetic robotics [55].

This chapter outlines the theoretical foundations and structural characteristics of such AI algorithms and classifies their technological applications in biomimetic robotics from a functional perspective. Each algorithm possesses distinct advantages depending on environmental conditions, data characteristics, and control objectives, and contributes in different ways to realizing the sensory integration, flexible motion control, and autonomous learning capabilities demanded by biomimetic robots. Therefore, the discussions in this chapter aim not only to provide a deeper understanding of AI technologies, but also to establish a theoretical basis for understanding how these technologies are being integrated into intelligent behavior generation in real robotic systems.

Thus, artificial intelligence is now actively utilized in biomimetic robots and plays a key role in controllers across various industrial fields, including automotive, aerospace, manufacturing, and energy. These algorithms can be categorized according to their learning paradigms—such as machine learning, deep learning, reinforcement learning, and evolutionary algorithms—with detailed theoretical backgrounds provided in the supplementary information. In this section, as summarized in Table 1 and Table 2, we focus on general examples of AI-based control, classifying the application cases in controllers according to reinforcement learning, deep learning, and evolutionary algorithm-based control methods.

### 3.1. Reinforcement Learning–Based Control Methods

Robotic manipulation control is one of the domains in which RL has been most actively applied. Robotic manipulation control can be broadly divided into machine learning–based classification/regression techniques(KNN, SVM, Random Forest, etc.) and deep reinforcement learning (Deep RL) using policy networks (cf. Figure S1). DRL can be used to train optimal control policies for robots performing complex manipulation tasks. One notable study by Google involved training hand–eye coordination for robotic grasping. In this work, multiple robots collected over 800,000 grasp attempts over two months. The collected data were used to train a controller capable of operating across uncalibrated robots with varying camera positions and gripper wear conditions. Additionally, over 900,000 grasp attempts were collected on another platform to test inter-robot transfer, demonstrating that combining data across robots leads to more reliable and effective grasp strategies [56].

DRL has also shown promising results in the fluid dynamics domain, particularly in active flow control (AFC), where control inputs are used to alter flow characteristics. DRL was successfully applied to stabilize the dynamics of chaotic systems governed by the Kuramoto–Sivashinsky equation, as well as to reduce skin-friction drag in wall-bounded channel flows. Notably, in channel flow experiments, DRL achieved a drag reduction rate of 46%, significantly outperforming the 20% achieved by traditional opposition control methods. The PPOalgorithm showed comparable performance to adjoint-based methods under low Reynolds number laminar flow conditions, while DDPG demonstrated effectiveness in handling high-dimensional action spaces [57].

In the context of Industry 4.0 (I4.0), DRL has also been applied to production planning and control (PPC), especially in production scheduling tasks. In resource planning problems, value-based or actor–critic methods such as Q-learning, Double Deep Q-Network (DDQN), and PPO have been used. One study proposed an optimization model using Q-learning, where the action was to determine the number of workers to hire based on the final inventory/surplus level and worker skill levels (state), with the goal of minimizing associated costs (reward). Another case applied policy iteration–based RL to control the operation of a two-machine production system with an intermediate buffer. The system determined whether to turn machines on or off based on machine failure status and buffer levels, aiming to reduce production costs, penalties for permanent production loss, and inventory holding costs [58].

In semiconductor supply chains, a DQN-based DRL framework was used to dynamically select the optimal demand forecasting model (action) based on historical inventory levels, shortages, past demand, and periods of zero demand (state). The system was trained to minimize forecasting errors (reward). In production scheduling tasks, value-based DRL methods such as DQN and Deep Q-learning, as well as policy-gradient and actor–critic methods (e.g., A2C, TRPO, PPO) have been applied. For instance, TRPO and PPO were applied to Job Shop Scheduling Problems, which aim to optimize the total time required to complete all jobs [58].

### 3.2. Deep Learning–Based Control Methods

Rather than separating feature extraction, selection, and classification into distinct stages, end-to-end deep learning models shown in Figure S2a learn a single mapping from raw images to steering commands. Researchers at NVIDIA trained a CNN that directly maps raw pixel data from a single front-facing camera to steering commands. The CNN architecture used corresponds exactly to the prototypical structure shown in Figure S2b. This represents an end-to-end learning approach and has proven to be remarkably powerful. The system learns internal representations for essential tasks such as detecting relevant road features, using only human steering angles as training signals—without any explicit training to detect road edges. Unlike conventional methods that explicitly break down the problem into lane detection, path planning, and control, this end-to-end system optimizes all processing steps simultaneously. The researchers argue that this results in better performance and smaller systems, as the internal components self-optimize for overall system performance rather than optimizing intermediate human-defined criteria (e.g., lane detection). The training dataset consisted of single images sampled from video and corresponding steering commands (represented as the inverse turning radius, 1/r). To teach the system how to recover from mistakes, the training data were augmented with additional images that simulated the vehicle being off-center or rotated with respect to the road direction. This augmentation was performed via perspective transformations on the images. During training, the system compares the steering command predicted by the CNN to the desired command for each image and adjusts the CNN weights using backpropagation. In this way, the trained model can generate steering from video images of a single center-mounted camera [59].

Another notable example uses the Direct Perception approach. Instead of parsing the entire scene or directly mapping images to driving actions, this method estimates key affordance indicators related to driving, i.e., environmental cues that are directly relevant to driving decisions. The approach maps image input to a small number of affordance indicators, such as the vehicle’s angle relative to the road, distances to lane markings, and distances to vehicles in the current and adjacent lanes—a total of 13 defined indicators. This strategy lies between mediated perception (e.g., lane detection) and behavior reflex (end-to-end), providing a balanced level of abstraction.

In the study, a deep CNN was trained to map input images to these semantically meaningful indicators, forming a compact but informative affordance representation. The researchers demonstrated that a simple controller using only the estimated vehicle position and heading could make high-level driving decisions and control the car smoothly. The objective was to minimize the lateral distance between the vehicle’s current position and the lane centerline. The system was tested in the driving simulator TORCS, and was quantitatively compared with several baselines, including a behavior reflex ConvNet, a mediated perception system using lane detection, and a GIST-based direct perception model. The ConvNet-based system significantly outperformed the GIST-based system and performed comparably or better than a DPM-based mediated perception approach on the KITTI dataset [60].

### 3.3. Genetic Algorithm–Based Control Methods

In one study, a Genetic Algorithm (GA)-tuned Adaptive Neuro-Fuzzy Inference System (ANFIS) controller was proposed for path-tracking control of unmanned aerial vehicles, specifically quadrotors—mechanically simple but highly nonlinear systems. The objective was to use GA to facilitate convergence toward optimal parameters in the ANFIS controller, thereby reducing learning error and improving control quality. The quadrotor was modeled using Newton–Euler equations, and a 3-DOF model was developed for flight in a two-dimensional vertical plane, with control inputs defined as thrust ($u_{1}$) and pitch moment ($u_{2}$).

ANFIS combines neural networks and fuzzy systems in a hybrid structure that defines membership functions (MFs) for fuzzy input variables and applies fuzzy rules to compute outputs. In this ANFIS-GA design, the core task is to select high-performing MFs. GA was used to automatically optimize the parameters of input and output fuzzy MFs in the ANFIS controller. In GA, each individual (chromosome) encodes MF parameters as real values and evolves to find the optimal combination. The objective function minimized the error between system output and desired output, specifically minimizing mean squared error (MSE) and root mean squared error (RMSE). Simulation results showed that the proposed ANFIS-GA controller outperformed both conventional PID and ANFIS controllers, minimizing error and enabling the quadrotor to reach target paths in a shorter time [61].

Another study implemented a fuzzy logic–based position controller on a real experimental platform (3D printer base), using a genetic algorithm to optimize the MF values for the controller’s input variables. Traditional controllers tend to underperform in time-varying systems, whereas AI-based methods can overcome these limitations and also reduce human error in fuzzy controller design. In this study, the primary role of GA was to optimize the fuzzy controller’s membership functions, specifically the parameters of triangular MFs used for the error variable. Each individual in the GA population consisted of a combination of parameters for these three triangular functions. An initial population was randomly generated, and individuals were evaluated using a fitness function based on the error between the desired value and actual output. Parents were selected and subjected to genetic operators such as crossover and mutation to produce offspring for the next generation. The process repeated until stopping criteria were met. The optimized MFs were then used to convert numerical values to linguistic representations during the fuzzification stage.

Experimental results across four scenarios (e.g., changes in weight or path) showed that the proposed controller reached the desired position despite changes in initial conditions. Notably, it outperformed comparable technologies in terms of rise time and settling time [62].

Lastly, a study proposed a GA-based automatic PID tuning method. While PID controllers are widely used in industry, tuning them remains a critical challenge for plant stabilization. The method is based on the hypothesis that any plant can be approximated by a second-order function, and proposes a novel auto-tuning technique. Time-domain parameters such as maximum overshoot and peak time are extracted from the plant’s unit step response to calculate the damping ratio and undamped natural frequency, which are then used to estimate an approximate second-order transfer function.

**Table 1:** Systematic Comparison of Representative AI Algorithms, Key Strengths and Weaknesses, and Control Applications.

Algorithm Type	Representative Algorithms	Key Strengths	Weaknesses	Application in Control Methods	Ref.
Machine Learning	KNN, SVM, Decision Tree, Random Forest, ANN	- Simple structure (KNN) - Strong classification performance in high dimensional space (SVM) - High accuracy, provides feature importance (RF) - Able to learn complex relationships (NN)	- Increased computational cost with large datasets (KNN) - High training time and memory requirements (SVM) - Prone to overfitting (RF) - Requires careful tuning and large datasets (NN)	Environment perception and prediction, data-driven state classification, sensor data-based fundamental decision-making	[48,63,64,65]
Deep Learning	CNN, RNN, LSTM, GRU, Transformer, CLIP, BLIP-2	- Strong for image and local pattern extraction, easy parallelization (CNN) - Superior in handling time-series/sequential data (RNN) - Learns long-term dependencies and bidirectional context (LSTM/GRU/BiLSTM) - Stable for time-series inputs (TCN) - Excellent for parallel processing and context understanding; scalable for large text/multimodal data (Transformer) - Universal image-language integration (Vision Transformer/CLIP/BLIP-2)	- Limited past information reflection, risk of overfitting (CNN) - Gradient vanishing with long sequences, difficult to parallelize (RNN) - Increased complexity and tuning burden (LSTM/GRU) - Sensitive to network design (TCN) - High computation/memory burden, prone to overfitting (Transformer, large multimodal models)	Vision-based control, navigation, voice command processing, vision-based behavior generation	[59,60,66,67,68,69,70,71,72,73]
Genetic Algorithms	GA, NEAT, HyperNEAT, GP-based NEAT	- No need for differentiation (GA) - Simultaneous optimization of network structure and weights, supports incremental complexity (NEAT) - Efficient evolution for large scale neural networks (HyperNEAT) - Rapid evolution, efficient architecture search (RBF-NEAT)	- High repetitive computation, slow convergence (GA) - Large computational cost for large networks (NEAT) - Complex indirect encoding design (HyperNEAT) - Requires tuning, burden of hybridization (RBF-NEAT)	Co-evolution of robot morphology and control strategy, neural network architecture optimization, PID/ANFIS tuning	[61,62,74,75,76,77,78,79]

GA is then applied to tune the PID gains based on this approximated model. The genome of each GA individual consists of $K_{p}$, $K_{i}$, and $K_{d}$. Fitness evaluation is based on minimizing the error between the system output and an ideal reference output approximated by a unit step response with low overshoot and ripple. Because the method uses an approximate model, it minimizes physical stress on the plant during tuning. To validate the method’s effectiveness, it was compared to MATLAB’s pidtune function. Comparisons based on rise time and settling time showed that the GA-based method generally achieved better results, especially in terms of rise time. The method has the advantage of requiring no prior knowledge of the control system and avoids complex mathematical modeling [74]. Additionally, the basic operations and descriptions related to RL, DL, and GA are all described in the supplemenatry.

**Table 2:** Systematic Comparison of Representative AI Algorithms, Key Strengths and Weaknesses, and Control Applications.

Algorithm Type	Representative Algorithms	Key Strengths	Weaknesses	Application in Control Methods	Ref.
Reinforcement Learning	DQN, PPO, TRPO, A3C, SAC, TD3	- Effective in discrete state spaces, supports experience replay (Q-learning/DQN) - Efficient parallel training and high sample efficiency (A2C/A3C) - Stable convergence with policy-based methods, suitable for continuous control (TRPO/PPO) - Off-policy; excels in continuous/high-dimensional control, good exploration-exploitation balance (SAC/TD3/DDPG) - High energy efficiency, fast learning with SNN integration (DRL + SNN)	- Limited in continuous/complex environments (Q-learning/DQN) - Requires reward design and parameter tuning (A2C/A3C) - High computational load, sensitive to parameter setting (TRPO/PPO) - Performance heavily affected by environment/reward design, tuning difficulty (SAC/TD3/DDPG) - Hardware deployment complexity (DRL + SNN)	Autonomous locomotion, underwater/aerial robot path optimization, robotic manipulation control	[54,57,58,64,80,81,82,83,84,85]

## 4. AI-Based Control of Biomimetic Robots

Biomimetic robots are designed to imitate organisms in nature, such as animals and insects, and are characterized by their enhanced flexibility, adaptability, and cooperativity. However, it is difficult to control such systems—often exhibiting complex and nonlinear dynamics—using only conventional algorithm-based control. In this context, AI models, particularly machine learning and deep learning, create strong synergy due to their ability to handle nonlinearities through data-driven learning. For example, biomimetic robots frequently involve multi-joint structures, elastic materials, and fluid joint configurations that are difficult to analytically model. Deep learning and reinforcement learning can improve control policies through data without requiring exact mathematical modeling, thereby reducing the burden of modeling complex structures and environments.

Moreover, the ultimate goal of biomimetic robots is to autonomously adapt to unknown environments like real animals. Reinforcement learning enables robots to learn autonomously through simulation or real-world experiments and discover optimal behaviors. In addition, biomimetic robots may be equipped with various types of sensors such as vision sensors, proximity sensors, and force/tactile sensors. Deep learning facilitates the integration and learning of such multimodal sensor data, enabling more precise control decisions. In this way, AI provides learning, inference, and optimization capabilities, allowing biomimetic robots to improve autonomy even in highly dynamic and complex environments. This represents a shift from traditional scripted robots to a hybrid paradigm, where robots continuously learn and discover optimal actions even in novel scenarios.

In Section 2, biomimetic robots were functionally classified into four major categories: (1) Locomotion imitation, (2) Sensory imitation, (3) Cognitive and learning imitation, and (4) Structural and regenerative imitation. Recently, AI technologies have been actively introduced across each of these domains. In this section, we review the literature and analyze how artificial intelligence has been applied in each functional area, including the specific control strategies adopted.

### 4.1. AI for the Control of Locomotion-Mimicking Biomimetic Robots

Biomimetic robots that imitate locomotion behaviors—such as animal walking, jumping, swimming, and body deformation—can demonstrate excellent mobility and adaptability in complex tasks, including human–environment interaction and exploration in extreme environments. By applying AI-based control techniques to these robots, their ability to respond to external sensor data or perform predictive motion has been significantly enhanced.

In this section, we provide an integrated analysis of the structures and characteristics of AI algorithms applied to locomotion-mimicking biomimetic robots, along with the control mechanisms enabled by these algorithms.

#### 4.1.1. Walking and Running Imitation

The exoskeleton robot FUM-Hexa, which imitates human walking, is a wearable system that actively controls joints by recognizing the user’s motion intention in real time through various biosignal-based sensors such as EMG, IMU, and FSR. In this study, the 99-dimensional features extracted with a 30-step sliding window were rearranged into a 10 × 10 lattice, enabling a convolutional neural network (CNN) to estimate the current joint position from multimodal sensor data. A two-layer bidirectional LSTM (250 × 250 units) subsequently predicted joint angles 67 samples ahead (near 134 ms), and these predictions were injected as the desired angle θ of an impedance controller. In the predictor-selection experiment, the BiLSTM achieved the lowest error, reducing the prediction error by 32–60% relative to the GRU. When the same network was embedded in the impedance-control loop, the joint-torque-tracking NRMSE fell to 0.012–0.092 (minimum 0.021), corresponding to a control-error reduction of up to ~70% compared with the 0-step baseline without prediction(~0.07–0.30). Overall, the CNN + BiLSTM predictive impedance control markedly outperformed conventional deep-learning variants (LSTM, GRU) and non-predictive impedance control, yielding superior tracking accuracy and human–robot synchronization across all participants and walking speeds [86].

Similarly, a controller was proposed for a 3-DOF bipedal robot leg, which integrates a DNN-based prediction model into an Model Predictive Control(MPC) framework. A deep learning model trained with TensorFlow was used as the model predictor, and this was embedded within the MPC optimization routine to calculate optimal torques in real time. The system’s convergence was guaranteed based on Lyapunov stability theory, During joint-trajectory tracking generated by a Gaussian process, nonlinear model predictive control (NMPC) achieved a trajectory mean-squared error of only 2 × $10^{-4}$ rad^2^. By contrast, the PID controller incurred much larger tracking errors and produced torques that surged to 100 Nm, repeatedly violating the system’s limits. Accordingly, NMPC delivered more than an order-of-magnitude improvement in tracking accuracy relative to classical PID while respecting torque constraints, thereby enhancing real-time, stable trajectory control of the robotic leg [2].

At the hardware level, control systems utilizing EAs have also been investigated. In an FPGA-based walking robot controller, each leg was controlled by circuits automatically evolved using a genetic algorithm and developmental encoding method. Each leg independently performed local control based on its own sensor data. This approach enabled cooperative walking through parallel operation even without neural network-based control and allowed for flexible adaptation to variations and complexities in body structure [9].

Recent studies have demonstrated the successful application of reinforcement learning (RL) methods directly to quadruped robot hardware, enabling effective locomotion even in complex natural environments. For instance, Lee et al. successfully deployed deep reinforcement learning (DRL) policies onto the hardware of the ANYmal robot, achieving stable locomotion across diverse terrains such as slippery slopes, rocky areas, and sandy surfaces [87]. Similarly, Bohlinger et al. utilized a highly efficient off-policy RL algorithm called CrossQ [88], obtaining a walking policy capable of speeds up to 0.85 m/s after only eight minutes of on-robot training, thus enabling rapid and robust locomotion across various challenging conditions [89]. These findings highlight the significant potential of RL-based control for practical deployment on real robotic systems.

#### 4.1.2. Swimming and Underwater Locomotion Imitation

**Figure 5:**
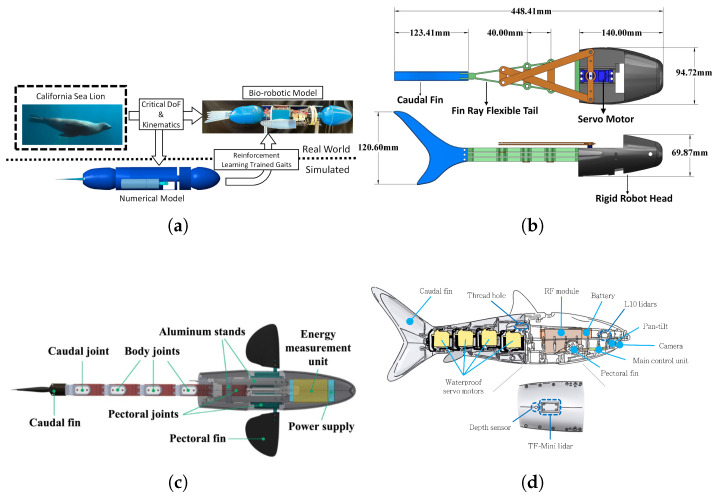
Biomimetic robots for underwater locomotion control: (**a**) Sea lion imitation robot (adapted from [90]). (**b**) Catfish imitation robot (adapted from [91]). (**c**) Fish motion imitation robot (adapted from [92]). (**d**) Shark imitation robot (adapted from [5]).

Robotic systems inspired by various aquatic animals such as fish, sea lions, and eels involve high-dimensional fluid–structure interactions, and reinforcement learning-based algorithms have been actively applied to control these systems. As shown in Figure 5, various robots that imitate underwater locomotion can be observed.

The SEAMOUR robot, modeled after the California sea lion, learned swimming motions using the SAC algorithm. Spline control points for roll, pitch, and yaw were defined as the action space, and a reward function integrating swimming distance, posture stability, and vibration minimization was used for training. The learned trajectory was deployed offline to the physical robot, and its performance was benchmarked against a biological reference trajectory—the Characteristic Stroke—that faithfully reproduces the fore-flipper motion of a real sea lion. Experimental trials demonstrated consistent advantages: forward travel distance increased by 7%, mean velocity by 8%, while cumulative pitch was reduced by 60%. Collectively, these results indicate superior straight-line propulsion and enhanced postural stability compared with the original natural motion [90].

A soft-bodied robot mimicking an eel performed online reinforcement learning also using SAC to discover its rotational cycle and direction autonomously, thereby maximizing forward velocity while simultaneously minimizing energy expenditure and lateral drift. Training was first conducted in a MuJoCo-based simulation for roughly 20,000 steps (near 5 h) and the resulting policy was transferred directly to the physical platform, achieving “online control” in real time. The torque waveform of a single cycle obtained from this online control was then approximated by a polynomial curve to create a fixed-period signal, termed “central offline control”. Two additional open-loop baselines (“offline 1” and “offline 2”) were generated by phase-shifting this signal by ±0.2 s. Straight-line trials were performed in a 1.8 m water tank for 8 s. The online controller covered 0.40 m, outperforming the central offline controller (0.36 m) by 11% and offline 1 (0.21 m) by 90%. Its fuel-specific efficiency index was 68.9, representing a 41% reduction in energy consumption relative to offline 1 (117.2). Moreover, the mean lateral deviation was only 0.014 m—an 85% improvement over offline 1 (0.093 m)—demonstrating quantitatively superior straight-line stability during swimming [93].

In another case, a soft magnetic microrobot(HAMR) inspired by helical propulsion mechanisms was controlled using the SAC algorithm without an explicit environment model. Within a rotating magnetic field generated by electromagnetic coils, the robot mimicked the asymmetric flagellar rotation of bacteria to move forward. SAC was used to learn a 4D continuous control vector. Training was performed in a physical test environment, and a continuous swimming policy was acquired within 100,000 steps along a clockwise trajectory. The learned policy could be approximated by mathematical functions (e.g., sine, square wave), improving execution speed and reliability. It achieved a forward velocity more than twice that of conventional random or offline waveform–based control strategies, while also revealing room for further enhancement through regression-based optimization. This models control strategy significantly expanded the applicability of soft microrobots where dynamic modeling is difficult [25].

In addition to SAC-based scheme detailed above, The SoftCon system, which mimics the flexible swimming of octopuses, jellyfish, and stingrays, integrated the PPO algorithm with a biologically inspired muscle stimulation model (AP-MEM). The system controlled contraction forces at each node via an FEM mesh, allowing the robot to autonomously learn target-directed motion and optimize propulsion in complex underwater environments. The average reward combined four terms: the error between target and actual speed, the angular deviation between the desired and actual heading, the pitch required to keep the body level, and several auxiliary objectives. This composite metric served to benchmark four controllers—AP-MEM, Independent-MEM (I-MEM), Shared-MEM (S-MEM), and Central-Pattern-Generator-MEM (CPG-MEM). In the eight-legged octopus model, AP-MEM achieved an average reward of 1503, whereas I-MEM and S-MEM reached only 8.6 and 15.1, respectively; the new method therefore improved performance by up to a factor of 175. Training time also fell from 156 h with I-MEM to 47 h with AP-MEM [94].

For the lamprey robot, AP-MEM reproduced biologically realistic swimming in 19 h, while I-MEM required 350 h, representing an eighteen-fold acceleration. In the starfish robot, AP-MEM obtained a reward of 1684 after just 6 h; this is thirty-three times faster than I-MEM and still preserves 92 percent of its final reward of 1809. Taken together, integrating AP-MEM with deep reinforcement learning yields almost two orders of magnitude better propulsive performance and more than an order-of-magnitude faster convergence than existing MEM-based approaches, especially in morphologically complex soft-robot systems [94].

Another study compared PPO, A2C, and DQN algorithms on a soft–rigid hybrid robot inspired by the pangasius fish. Whereas PPO rapidly converged to a mean episode reward of approximately 200, A2C and DQN plateaued at roughly 160 and 120—25–40% lower—so the consistently superior stability of PPO led to its adoption for the final implementation. The tail stroke cycle was set as the control variable, and direct training was performed in a physical environment, naturally incorporating system nonlinearity into the control strategy [91].

For a multi-joint robotic fish utilizing the carangiform swimming pattern, a cooperative control framework combining open-loop Central Pattern Generator(CPG)-based control with closed-loop DDPG correction was proposed. A Hopfield-based MLP generated rhythmic swimming control parameters, while DDPG provided real-time adjustment, improving both energy efficiency and swimming stability In a single-subtask simulation, CSC reduced energy dissipation by 60.6% relative to the previous optimum and raised the target-zone success rate from 51.2% to 90.4%, representing a 76.6% improvement. Subsequent water-tank trials showed that CSC lowered electrical energy consumption by 38.72%, 22.13%, and 23.97% compared with PID, ADRC, and SMC controllers, respectively, while still tracking the task accurately. These findings indicate that CSC simultaneously enhances energy efficiency and trajectory-generalization performance [92].

#### 4.1.3. Morphing Locomotion Imitation

The STIFF-FLOP continuum robotic arm, inspired by the structure of an octopus arm, employed the Dynamical Systems–Gaussian Mixture Regression(DS-GMR) algorithm to learn the curved reaching trajectories of octopus motion. Motion trajectories based on Euler angles were generalized using a Gaussian Mixture Model(GMM), and temporal motion sequences were predicted using Gaussian Mixture Regression(GMR). The system performed iterative self-refinement through reward-weighted regression, enabling average episode reward rose from 0.42 to 0.88—a 110% improvement—while the maximum tip error fell by 56%, from 41 mm to 18 mm. Consequently, the proposed control strategy achieved roughly twice the imitation accuracy of a straightforward policy transfer without self-refinement [4].

In modular robotic systems that autonomously assemble and move, an evolutionary algorithm based on the Artificial Homeostatic Hormone System(AHHS) was applied. Each module regulates its local state by generating, diffusing, and degrading hormones, and autonomously controls the connection, disconnection, and movement of the overall structure. Hormonal reaction rules were optimized through genetic algorithms, ensuring high flexibility in structural diversity and environmental adaptability. When the number of modules was increased from two to seven, the system regained 95% of its original speed within 25 generations, yielding caterpillar-like straight locomotion. In the T-shaped, three-limb robot, continued evolution shifted the initial “planar crawling” strategy to a tripod gait, raising the mean forward velocity by an additional 23% within the same simulation time. These results demonstrate strong robustness, enabling rapid recovery and even enhancement of performance despite increases in module count or morphological alterations [27].

AI-based controllers applied to morphing locomotion robots not only implement the physical mechanisms of biological motion strategies, but also replicate neural control structures, coordination patterns, and muscle responsiveness within digital control frameworks. In particular, reinforcement learning-based controllers provide flexible learning architectures that ensure autonomy and stability in complex nonlinear environments, effectively reproducing the adaptive motor abilities of biological organisms. Moreover, EAs and machine learning-based continuum control methods break away from rigid one-to-one mappings between morphology and control, suggesting the potential for generalization across diverse body structures.

Looking forward, such AI-based control technologies are expected to contribute to the realization of natural and adaptive motion similar to that of humans in a wide range of real-world applications, including exoskeletal assistive devices, underwater exploration robots, and soft surgical robots.

### 4.2. AI for the Control of Sensory-Mimicking Biomimetic Robots

Biomimetic robots that imitate sensory functions enable sensitive responses to external environments, recognition of object properties, and autonomous adaptation to dynamic situations. Biological sensory systems—such as vision, hearing, and touch—possess intricate architectures that simultaneously achieve high-dimensional information processing and rapid responsiveness. To replicate these capabilities, a variety of AI algorithms have been applied to sensory-mimicking robotic systems.

This section analyzes the application of deep learning, EAs, and SNN-based control methods in the control of sensory biomimetic robots, and describes how each approach has been used to reproduce specific characteristics of biological sensory systems.

#### 4.2.1. Vision Imitation

In a visual cognition system that mimics the function of the human visual cortex, an HMAX-based hierarchical structure was augmented with memory and associative mechanisms to improve interpretability and precision in object recognition. The model discriminates input objects by dual comparison of episode-based similarity and semantic-based attributes. After visual feature extraction, activation patterns are generated in a form analogous to biological neural activity. Control is achieved via a higher-level inference module that performs semantic similarity alignment and reasoning based on visual features extracted from the S1–C2 layer. In a few-shot face-recognition setting with only five images per subject, the proposed model—using just two facial regions comprising eight patches—achieved an accuracy of 90.77%, a 28-percentage-point improvement over RandPatch-HMAX under identical conditions (70.7%), while its reduced patch count also conferred greater memory efficiency [95].

In a fully physiology-based visuomotor integration system, a neural network-based controller was proposed to regulate the muscles of the eyes, head, arms, and legs in response to visual stimuli. Visual information is processed through a DNN-based cortical module, while a motor DNN controls more than 284 muscles via both voluntary and reflexive pathways. Each DNN receives integrated input from gaze direction, positional error, and muscle feedback, and outputs contraction/relaxation signals to the muscles. This enables the robot to perform complex behaviors such as handwriting, object catching, and visual tracking [96].

In a biomimetic robotic head controller specialized for gaze tracking, a SNN mimicking the anatomical connectome of the human oculomotor system was applied. Even without training, neuronal firing patterns are triggered in response to visual stimuli, which are converted into servo motor commands that drive rotational movement of the eyes and neck. A Hebbian reward-based learning rule was additionally applied to enhance vertical tracking precision and reduce convergence time. Comparing results before and after the rule addition, the root-mean-square error (RMSE) of the left-eye horizontal channel decreased from $3.55^{\circ }$ to $2.87^{\circ }$, and the relative error (RE) improved from $-1.87^{\circ }$ to $-1.49^{\circ }$, corresponding to an about 19% improvement and confirming an overall error reduction of roughly 15% [97].

In the robotic lamprey system, visual stimuli were processed by a neural network composed of leaky integrate-and-fire (LIF) neurons, using both event-based and frame-based camera data. The system determined stimulus priority and generated motor commands, delivering signals to the CPG to control turning angles and propulsion speeds. Even in situations involving competing stimuli, the robot demonstrated appropriate direction tracking and avoidance behavior. This structure represents a numerical implementation of a central nervous system-based controller that links the visual cortex to the brainstem. Compared with deploying the same network on a frame-based camera, the cumulative gaze misalignment with the target decreased by approximately 83%. In addition, the neuron operation rate (passes s^−1^) rose by at least one order of magnitude—shifting from the single- to the double-digit logarithmic range across all test conditions—thereby markedly enhancing real-time responsiveness [98].

#### 4.2.2. Tactile Imitation

**Figure 6:**
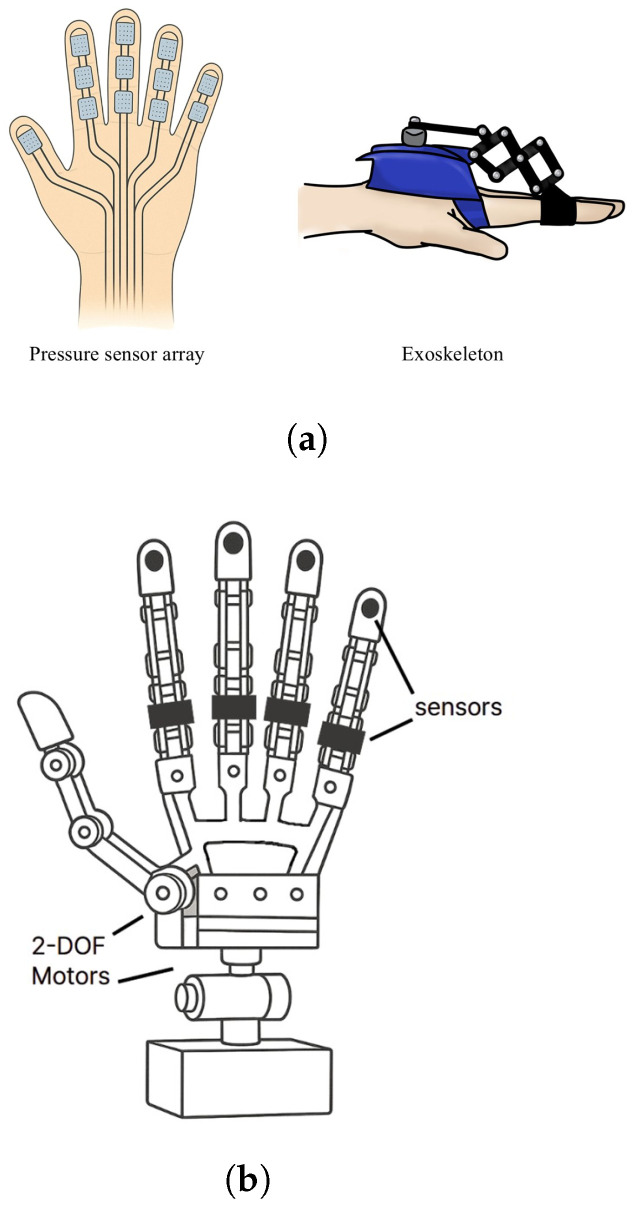
Tactile imitation-based robotic hand control systems: (**a**) Pressure-sensing gloves and exoskeleton systems (adapted from [99]). (**b**) Tendon-driven robotic hand learning grasping strategies through self-exploration (adapted from [100]).

In a robotic hand system that mimics pressure-based grip control in the human hand, a Recurrent Convolutional Neural Network(R-CNN) was used to process spatiotemporal pressure data. The CNN extracts features from the pressure distribution on the finger surface, while the RNN learns temporal variations to predict the timing of grip completion. The prediction results are used to adjust the force of the robotic hand and to determine the completion of the grip, achieving a high prediction accuracy of 77 84.5% across various objects. This system can be applied to medical prosthetic hands and industrial robotic grippers (Figure 6a), and implements biomimetic tactile control through a real-time sensing–control loop [99].

In another robotic hand control system that learns grasping strategies through self-exploration, a neural controller based on an evolutionary algorithm was applied (Figure 6b). Synaptic connection rules inspired by biological ligand–receptor mechanisms were used, and neuron weights were updated based on reward signals derived from pressure sensors. The neurons controlled finger motion in response to sensory input, and neural networks that achieved higher rewards were passed on to subsequent generations through evolution. This represents an example of a self-adaptive learning control architecture based on tactile feedback rather than simple repetition [100].

#### 4.2.3. Acoustic and Vibration Sensing Imitation

**Figure 7:**
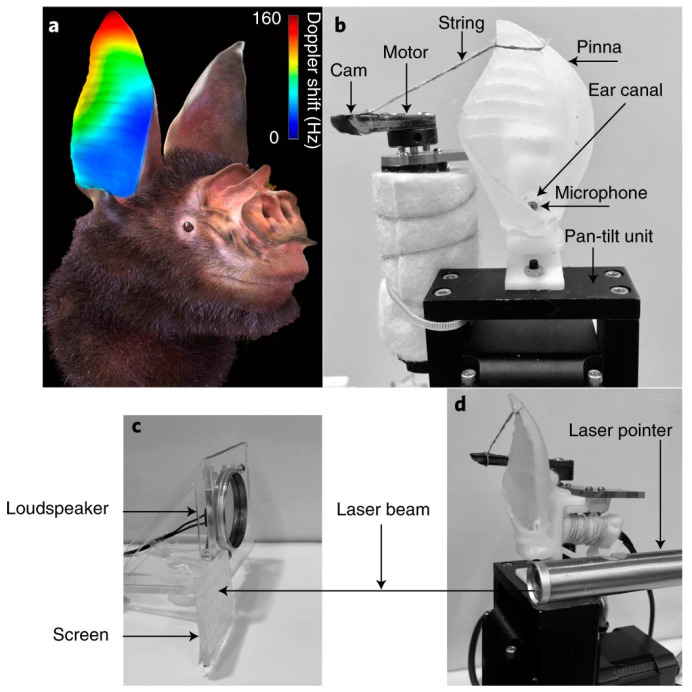
Acoustic and vibration sensing imitation with a bat-inspired ear structure. (**a**) Complex Doppler-shift distribution generated by dynamic deformation of the real pinna surface of Pratt’s roundleaf bat; (**b**) Silicone-based biomimetic robotic pinna and its experimental setup; (**c**) Frequency-generating speaker and alignment screen used in the experiment; (**d**) Biomimetic pinna equipped with a laser pointer to verify its orientation (adapted from [101]).

Figure 7 shows a robotic system that mimics the bat’s ability to detect sound direction by using a biomimetic soft pinna to enable directional sensing with only a single microphone and a single frequency. A CNN was used to process the modified ultrasonic spectrum, which was affected by the Doppler effect, allowing for high-precision direction estimation with a mean error of just 0.48°. A motor–cam mechanism was employed to deform the pinna, and the CNN output was used to align a laser pointer for real-time direction tracking in an integrated control structure. This represents a notable example of achieving high performance by combining dynamic structural modulation with AI-based interpretation, without relying on fixed sensor arrays [101].

### 4.3. AI for the Control of Cognitive and Intelligence-Mimicking Robots

The cognitive and learning abilities of animals play a crucial role in adapting to their environments and making appropriate decisions depending on the situation. Biomimetic robots that emulate these abilities can go beyond simple reactive control to implement higher-level intelligence, including experience-based behavioral adaptation, implicit learning without feedback, and integrated processing of sensory and motor information.

This section analyzes how AI algorithms that imitate biological cognition and learning strategies are being applied to robotic systems, and how these approaches are being implemented through specific control methodologies.

#### 4.3.1. Adaptive Neural Imitation

In a neural activity-based path generation and control system, a neural field model based on the Hodgkin–Huxley membrane model and Grossberg’s shunting model was proposed. The target position is modeled as an excitatory peak, while obstacles are modeled as inhibitory valleys, allowing the robot to generate paths according to the gradient of activation. This system is an unsupervised neural dynamic control approach that does not require learning and is applicable to both mobile and multi-joint robots. It integrates real-time path regeneration, obstacle avoidance, and target tracking functionalities, and system convergence and stability were theoretically verified via Lyapunov-based stability analysis [102].

In a study mimicking the implicit learning processes of humans and animals, a distributed neural network based on an extended Hebbian rule was employed. Each neuron possesses a firing state and autonomously adjusts its threshold and synaptic weights in response to input stimuli. Without any external force or reward signals, the system self-learns complex behaviors such as joint motion, balance maintenance, and rhythmic movement generation. Through interconnections between neuron clusters, the system achieves both stability and energy efficiency. This is a representative case of a biomimetic adaptive learning controller that performs environmental adaptation without supervised or explicitly reinforced learning [103].

As an example of a biomimetic adaptive controller applied to a real robot, Human-like Compliant Movement Primitives(Hl-CMPs) were proposed to emulate the feedforward and impedance regulation capabilities of human muscle control. This system dynamically updates impedance and force parameters for each task axis based on Dynamic Movement Primitive(DMP)-generated motion trajectories and was validated in insertion and cutting tasks using the KUKA LBR iiwa robot. Experimental results revealed that the mean Z-axis tracking error decreased by roughly 40%, the contact-force ripple was attenuated by as much as 70%, and the lateral forces $F_{x}$ and $F_{y}$ were constrained to nearly 0 N, thereby preventing surface damage, successfully achieving more biomimetic adaptive motion than conventional fixed impedance control [104].

#### 4.3.2. Swarm Behavior Imitation

A representative case of transplanting biological swarm behavior into robotics is a study that implemented social interaction between a school of fish and a robotic agent. The robot was designed with a biomimetic appearance and received the position, velocity, and distance information of real fish as input. A Deep Learning Interaction (DLI) model produced the robot’s acceleration distribution, which was then used to generate a tracking trajectory via a PID controller. The robot autonomously performed swimming behaviors that maintained social distance and alignment, forming a group that closely resembled real fish. Both experiments and simulations demonstrated a high degree of behavioral conformity [105].

In another study, DNN was used to learn asymmetric inter-agent interactions from actual fish schooling data, and this model was applied to swarm robot control. Each robot used a regression DNN composed of an Adaptive Coordination Network (ACN) and Local Decision Network (LDN) to predict rotation direction, travel distance, and time. Decision-making was based solely on information from a single neighbor. Crucially, a Leader Vision Pressure Selection (LVPS) strategy was applied, in which only the agent occupying the widest field of view was chosen as the leader. This ensured both biological plausibility and computational efficiency. Experimental results showed that the model successfully reproduced real fish-like collective behaviors—such as alignment, cohesion, and counter-milling—in both simulation and physical robot experiments [106].

AI algorithms applied to cognitive and learning-mimicking robotic systems go beyond simple reactive or pre-programmed rule-based control, and instead emulate key functions of the biological nervous system—such as autonomous adaptation to environmental changes, learning without explicit feedback, and muscle-like interactive control. In particular, learning architectures without explicit rewards, nonlinear musculoskeletal adaptation, and neural network-based path planning are emerging as notable biomimetic intelligence frameworks that differ from conventional reinforcement learning–centered robot control approaches.

### 4.4. AI for the Control of Structural and Regenerative Biomimetic Robots

Biological organisms have developed various strategies—such as flexible adaptation, self-repair, and structural strength modulation—to maintain structural stability and functional continuity in the face of environmental changes or external impacts. Biomimetic robots that imitate these structural and regenerative capabilities incorporate composite materials, flexible connection mechanisms, and self-reinforcing systems, and require the application of advanced AI techniques, such as deep reinforcement learning, for effective control.

This section focuses on flexible structure imitation and self-healing imitation, and analyzes how each robotic system has integrated biomimetic structural characteristics with AI control algorithms to enhance control performance.

#### Self-Healing Imitation

In a biohybrid robot that incorporates a muscle use–based strengthening mechanism, the system mimics the biological characteristic where muscle force output improves through repetitive exercise. In a honeycomb structure composed of 42 distributed muscle units, the strengthening state of each muscle is updated based on its prior usage history and strain level. Using the PPO algorithm, the agent determines the activation level (0 to 1) of each muscle.

Control is implemented via a real-time feedback loop combining the PyElastica simulator and a Cosserat rod–based soft-body physics model, which calculates dynamic movement responses. Rewards are provided based on the agent’s ability to reach a target position. Strengthened muscles generate higher force output than non-strengthened ones, and the agent utilizes this adaptivity to reach targets more quickly and stably.

According to learning curve analysis, the adaptive agent initially demonstrated lower performance than the non-adaptive agent, but eventually achieved higher success rates and better energy efficiency in later stages. This indicates that the curriculum learning effect, analogous to biological muscle memory, contributed to improved training stability [44].

Table 3 systematically summarizes the specific control methods and structural characteristics, applied AI algorithms (including deep learning, reinforcement learning, and evolutionary algorithms), and concrete implementation features used in each functional domain (locomotion, sensing, cognition, structure, etc.) of biomimetic robots, based on the latest research. This table provides a comprehensive overview of how artificial intelligence is applied across various functions of biomimetic robots, as well as the distinctive control architectures and implementation features in each domain.

Thus, structural and regenerative biomimetic robots go beyond merely imitating external flexibility, and instead emulate biological strategies for sustainability, such as structural resilience, functional evolution, and movement-based adaptation. In particular, reinforcement learning–based controllers have proven effective for high-dimensional continuous control and multi-degree-of-freedom muscle activation, presenting a new intersection between controller design and biological evolutionary mechanisms by integrating structural feedback into the learning process.

## 5. Conclusions

As reviewed in this paper, integrating artificial intelligence technologies into biomimetic robots is not only essential but also provides significant advantages. Biomimetic robots are designed to mimic the sensory, learning, decision-making, and motor control abilities of animals to adapt to complex environments. However, traditional model-based control approaches face inherent limitations in effectively handling such high-dimensional, nonlinear behaviors. In this context, the adoption of machine learning and deep learning techniques enables robots to perceive and learn from their surroundings, granting them cognitive and inferential capabilities to interpret sensor inputs and make optimal decisions in real time. For example, deep learning–based multimodal sensor fusion integrates diverse sensory data to enable fine-grained control, while reinforcement learning algorithms allow robots to autonomously discover optimal control strategies through trial and error. As a result, AI integration enhances adaptability, efficiency, and accuracy, enabling higher-level behaviors such as predictive decision-making, sensor fusion perception, and uncertainty handling, far beyond what rule-based control can achieve. As demonstrated in examples of walking robots, aerial robots, swarm systems, and biohybrid devices, AI-based control serves as a key enabler in overcoming the functional limitations of biomimetic robots, facilitating more flexible and intelligent behavior that closely resembles biological organisms.

Looking ahead, the role of AI technologies in the field of biomimetic robotics is expected to expand further. In particular, reinforcement learning, including deep reinforcement learning, will continue to evolve as a core approach for enabling robots to learn optimal behaviors through experience in complex environments. Similarly, adaptive control and online learning methods will allow robots to adjust control parameters and respond to changes in real time based on sensory feedback, maintaining stable operation even in the face of unexpected disturbances or environmental variations. Additionally, neural network–based simulation and digital twin technologies will accelerate design optimization and control strategy development by enabling prediction and learning of robot behavior in virtual environments, thereby reducing real-world trial-and-error. These AI advances will further enhance real-time sensor feedback processing, improve responsiveness, enable energy-efficient control that emulates the economical movement of biological systems, and strengthen the robot’s ability to perceive and operate in diverse, unstructured environments. Furthermore, in terms of human–robot interaction, advanced AI will allow robots to more accurately understand and respond to human intent and behavior, enabling more natural collaboration and significantly advancing overall robotic autonomy. In sum, the convergence of AI and biomimetic technologies is expected to lead the future direction of intelligent robotics and usher in a new era of engineering adaptive biological intelligence.

## Figures and Tables

**Table 3:** Summary of AI-Based Control Methods, Applied AI Algorithms, and Implementation Features by Functional Domain in Biomimetic Robots.

Functional Area	Sub-Functional Area	Control Methods and Structural Characteristics	Applied AI Algorithms	Control Characteristics and Implementation Method	Ref.
Locomotion imitation	Human gait imitation	Predictive torque-based control	CNN + BiLSTM	Multi-sensor fusion, prediction-angle-based impedance control, network embedded	[86]
	3-DOF bipedal robot locomotion	DNN-prediction embedded MPC	DNN	Real-time torque optimization, Lyapunov stability, NMPC/PID performance comparison	[2]
	Multi-legged locomotion (evolutionary local control)	Evolutionary circuit-based distributed control	EL	Parallel operation, sensor-based local control, structural/contextual adaptation	[9]
	Quadrupedal locomotion	Direct control using reinforcement learning policy	Deep RL	Adaptation to various terrains in real environments, rapid learning, fast policy transfer	[87]
	Quadrupedal locomotion (high-efficiency off-policy RL)	CrossQ off-policy policy learning	CrossQ(Off-policy RL)	Rapid acquisition of walking policy within 8 minutes, fast convergence in real environment	[88,89]
	Sea lion swimming robot	Predictive trajectory-based roll/pitch/yaw control, biological motion benchmarking	SAC	Performance comparison with biological trajectories, offline training and policy transfer	[90]
	Eel-like soft robot	Online reinforcement learning-based propulsion control	SAC	Simulation-to-reality policy transfer, improved energy efficiency and straightness	[93]
	Helical propulsion microrobot	4D continuous control + policy function approximation	SAC	Real-world experiments, policy-to-function approximation, simultaneous improvement of speed and reproducibility	[25]
	Soft-bodied robot inspired by mollusks	FEM-based fluid–structure interactive propulsion control + PPO	PPO + AP-MEM	Composite rewards (target speed/angle), superior learning/performance to previous MEM	[94]
	Pangasius hybrid robot	PPO-based tail gait control	PPO, A2C, DQN	PPO superiority in stability and performance, inherent nonlinear characteristics	[91]
	Multi-jointed fish robot	CPG-based rhythmic control + DDPG closed-loop correction, improved energy efficiency and generalization	Hopfield MLP + DDPG	Cooperative control, experimentally validated energy savings and improved tracking performance	[92]
	Continuum robot (STIFF-FLOP)	DS-GMR-based trajectory generalization, self-compensating iterative learning	DS-GMR (GMM + GMR)	Trajectory generalization + self-compensation, more than twofold improvement in imitation through repetition	[4]
	Modular self-assembling robot	AHHS-based evolutionary structural contro	AHHS+EA	Rapid speed recovery and strategy switching with increasing modules, structural diversity ensured	[27]
Sensory imitation	Vision (cortical imitation)	HMAX hierarchical structure + associative memory control	HMAX + Memory/Association	Few-shot recognition, simultaneous improvement in efficiency and accuracy via patch reduction	[95]
	Visuo-motor integration	Visual-muscular integrated DNN control	DNN	High-dimensional input integration, spontaneous/reflexive actions, autonomous control of complex motions	[96]
	Visual tracking (oculomotor)	SNN-based anatomical circuit control	SNN + Hebbian reward	Improvement in pre/post-training performance and convergence time, real-time servo motor integration	[97]
	Vision-CPG (Central Pattern Generator) coupling	LIF SNN-based vision-priority and CPG-coupled control	LIF SNN	Visual stimuli linked to motion, tenfold increase in response speed over frame-based, reduced error	[98]
	pressure-based grip	R-CNN-based pressure time series control	R-CNN (CNN + RNN)	Real-time sensor-control loop, improved grip precision for medical/industrial applications	[99]
	self-exploratory grip strateg	Evolutionary neural network-based adaptive control	Evolutionary algorithm-based neural network	Self-adaptive control, self-learning based on pressure feedback	[100]
	Auditory/vibration (bat ear-inspired)	CNN-based Doppler acoustic control	CNN	High-precision direction sensing with single microphone/frequency, real-time dynamic control	[101]
Cognition and intelligence imitation	Neural dynamic path generation	Neural field-based path generation	Neural field model	Unsupervised real-time path planning/obstacle avoidance, Lyapunov-based stability verification	[102]
	Implicit learning	Distributed neural network-based threshold/weight self-adjustment	Extended Hebbian neural network	Complex motion/balance/generation without external rewards, improved energy efficiency	[103]
	Human-like muscle-compliant motion	DMP + impedance adjustment, compliant control with dynamic parameter update	HI-CMP (DMP + impedance)	Insertion/cutting tasks, reduced tracking error/force ripple, prevention of surface damage	[104]
	Fish school interaction imitation	DLI-based acceleration distribution prediction + PID tracking	DLI (DNN) + PID	Social distance/alignment behavior clustering in both real and simulated environments	[105]
	Asymmetric swarm DNN (Deep Neural Network)	ACN + LDN, LVPS-based single neighbor interaction	DNN (ACN + LDN) + LVPS	Biological plausibility + computational efficiency, implementation of alignment/cohesion/counter-milling	[106]
Structural and Regenerative imitation	Muscle-use reinforcement (self-reinforcement)	PPO-based distributed muscle activation control	PPO	PyElastica + Cosserat physical model, adaptive agent, curriculum learning effects	[44]

## Data Availability

Not applicable.

## Supplementary Materials

The following supporting information can be downloaded at: https://www.mdpi.com/article/10.3390/biomimetics10070460/s1, Figure S1: Structures of machine learning algorithms. (a) K-Nearest Neighbors (KNN). (b) Support Vector Machine (SVM). (c) Random Forest (RF). (d) Neural Network (NN); Figure S2: (a) Structure of deep learning. (b) Structure of a Convolutional Neural Network(CNN).

## Abbreviations

The following abbreviations are used in this manuscript:

DOF	Degree-of-Freedom
ACN	Adaptive Coordination Network
AFC	Active Flow Control
AHHS	Artificial Homeostatic Hormone System
AI	Artificial Intelligence
ANFIS	Adaptive Neuro-Fuzzy Inference System
ANFIS-GA	ANFIS-Genetic Algorithm
ANN	Artificial Neural Network
AP-MEM	biologically inspired muscle stimulation model
BCF	Body / Caudal Fin
BLIP	Bootstrapping Language-Image Pre-training
BMAI	Brain-Morphic Artificial Intelligence
BREAD	Biomimetic Research for Energy-efficient AI Designs
BSNN	Basic Spiking Neural Network
CGR	Climbing Gecko Robot
CNN	Convolutional Neural Network
CPPN	Compositional Pattern Producing Network
CPG	Central Pattern Generator
DL	Deep Learning
DLQR	Discrete-time Linear Quadratic Regulator
DOAJ	Directory of Open Access Journals
DQN	Deep Q-Network
DS-GMR	Dynamical-System Gaussian Mixture Regression
DSP	Digital Signal Processing
DRL	Deep Reinforcement Learning
EA	Evolutionary Algorithm
EMG	Electromyography
ETFE	Ethylene-Tetrafluoroethylene
FEM	Finite Element Method
FPGA	Field-Programmable Gate Array
FSR	Force-Sensing Resistor
FUM	Ferdowsi University of Mashhad
GA	Genetic Algorithm
GMM	Gaussian Mixture Model
GMR	Gaussian Mixture Regression
GPS	Global Positioning System
GRN	Gene Regulatory Network
GRU	Gated Recurrent Unit
HMAX	Hierarchical Model and X
HMM	Hidden Markov Model
I4.0	Industry 4.0
IMU	Inertial Measurement Unit
LIF	Leaky-Integrate-and-Fire
LSTM	Long Short-Term Memory
LVPS	Low-Voltage Power Supply
MDP	Markov Decision Process
MF	Membership Function
ML	Machine Learning
MLP	Multilayer Perceptron
MPC	Model Predictive Control
MPF	Muscle-Physiology-Inspired Fin
NMPC	Non-linear Model Predictive Control
NN	Neural Network
PID	Proportional-Integral-Derivative
PPO	Proximal Policy Optimization
PPC	Particle Swarm Optimization
PSO	Particle Swarm Optimization
PVDF	Poly-Vinylidene Fluoride
RBF-NEAT	Radial-Basis-Function NEAT
RF	Random Forest
RGR	Rigid Gecko Robot
RI	Reinforcement Learning
RL	Root-Mean-Square Error
RMSE	Root Mean Square Error
RNN	Recurrent Neural Network
RRT	Rapidly-exploring Random Tree
SAC	Soft Actor-Critic
SMA	Shape-Memory Alloy
SNN	Spiking Neural Network
STIFF-FLOP	STIFFness-controllable Flexible and Learnable manipulator for surgical OPerations
STOMP	Stochastic Trajectory Optimization for Motion Planning
SVM	Support Vector Machine
TCN	Temporal Convolutional Network
TORCS	The Open Racing Car Simulator
TRPO	Trust-Region Policy Optimization
UKF	Unscented Kalman Filter
UUV	Un-manned/Underwater Vehicle
VGG	Visual Geometry Group
VMP	Velocity-based Movement Primitive
