# Supplementary material for: AI-Driven Control Strategies for Biomimetic Robotics: Trends, Challenges, and Future Directions
Source: Biomimetics (Basel). 2025 Jul 14;10(7):460. doi: 10.3390/biomimetics10070460 (PMC12292875; doi:10.3390/biomimetics10070460)
Supplement: Supplementary file 1 [file biomimetics-10-00460-s001.zip › biomimetics-3672908-supplementary.pdf]

**Supplementary Materials**

## Sl. Machine Learning

Machine learning refers to a collection of algorithms that learn patterns from data without being explicitly programmed. Most machine learning algorithms train models using past data to make predictions on new data. Depending on the application method, machine learning is categorized into supervised learning (classification, regression), unsupervised learning (clustering, dimensionality reduction), semi-supervised learning, and reinforcement learning. Among these, **supervised learning classification algorithms** have been particularly well studied, with representative examples including **K-Nearest Neighbors (KNN), Support Vector Machine (SVM), Decision Tree**, its ensemble form **Random Forest (RF)**, and **Artificial Neural Network (NN)** . Each algorithm has different strengths and weaknesses depending on the nature of the data and the problem type, and according to the "No Free Lunch" theorem, no single algorithm is best for every problem.

K-Nearest Neighbors (KNN) is an instance-based classifier that classifies a new sample by comparing it with the closest training data points. It is simple to implement and intuitive, but since it stores and references all data during prediction without a training process, computational cost increases significantly as the dataset grows. Additionally, selecting the ideal number of neighbors $K$ is critical to performance but lacks a clear criterion and must be set empirically . Nonetheless, KNN serves as a useful baseline and performs reasonably well on small datasets(Figure S1a).

**Support Vector Machine (SVM)** is an algorithm that finds a maximum-margin hyperplane by mapping the input data into a higher-dimensional space. It is relatively resistant to overfitting, performs well in high-dimensional settings, and can handle non-linear problems via kernel functions. SVM is known to be robust against noise and imbalanced datasets, effectively avoiding overtraining, and can achieve high accuracy with a well-chosen kernel function. However, training time is often long, memory usage is high, and the resulting model is complex and difficult to interpret (Figure S1b).

**Random Forest (RF)** is an ensemble learning technique that constructs multiple decision trees from training data and outputs the final result via majority voting. Even if individual trees overfit, averaging over many trees cancels out the error, often leading to stable and high accuracy. RF is relatively less sensitive to outliers or noise and provides variable importance metrics. However, small changes in the training data can lead to different results, and models consisting of many trees are difficult to interpret. In some cases, overfitting has also been reported. Nevertheless, RF is widely favored alongside SVM for its high accuracy and ease of implementation (Figure S1c).

**Neural Networks (NNs)** are networks composed of nodes and weights that mimic the structure of neurons in the human brain, capable of learning complex non-linear relationships as universal function approximators. With sufficient data, NNs have high expressive power and potential for excellent performance. In particular, deep neural networks are highly effective for processing unstructured data such as speech and images. However, their complex architecture and numerous parameters require significant time and resources for hyperparameter tuning to find optimal weights and structures. They also demand large datasets, and selecting the optimal model architecture (e.g., layer types, activation functions) suited to the problem is challenging. For these reasons, despite their potential, neural networks are sometimes considered less practical, and algorithms like SVM or RF may be preferred when data are relatively limited (Figure S1d).

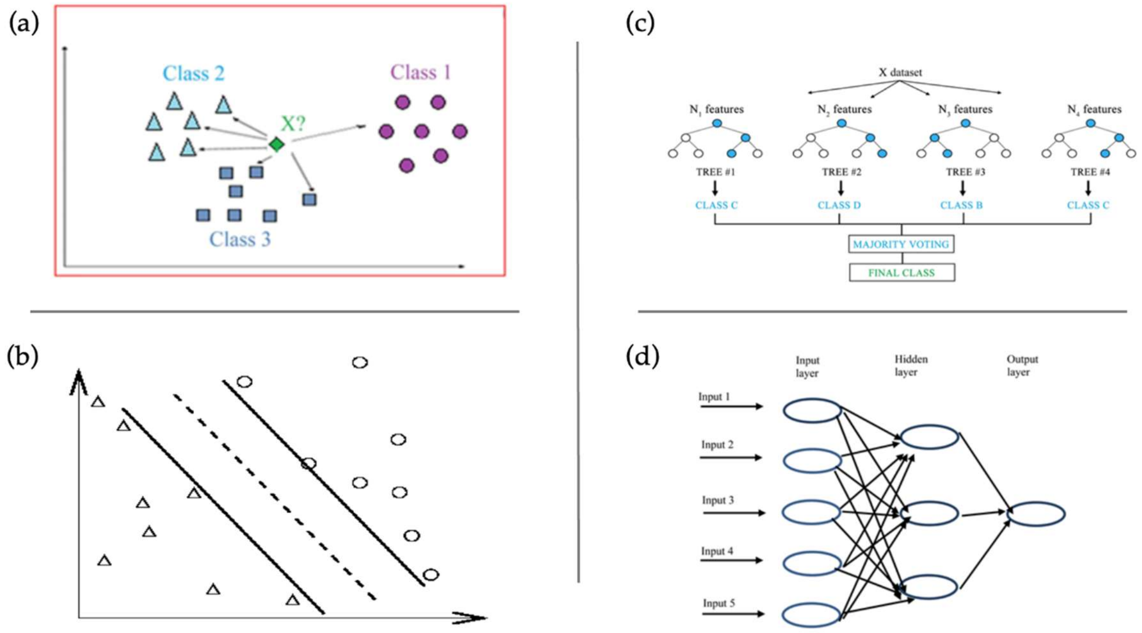


**Figure S 1. Structures of machine learning algorithms. (a) K-Nearest Neighbors (KNN). (b) Support Vector Machine (SVM). (c) Random Forest (RF). (d) Neural Network (NN).**

In summary, traditional machine learning algorithms each have distinct advantages and disadvantages and must be selected based on the characteristics of the problem. For example, in small datasets or problems with simple feature spaces, interpretable and easy-to-implement algorithms like decision trees or KNN may be suitable. In contrast, SVM and ensemble methods such as RF tend to perform more reliably on high-dimensional and complex data. Many studies point out that while SVM and RF achieve consistently high accuracy with minimal tuning, neural networks require more data and optimization effort. Data scientists typically compare these methods empirically to select the optimal model. For instance, in a study predicting mineral discovery potential in geophysical exploration using NN, RF, decision trees, and SVM, algorithm performance varied by data characteristics, but ensemble tree models and SVM generally showed stable accuracy.

Ultimately, machine learning often involves experimentally comparing multiple algorithms to obtain the best performance, and in recent years, there have been efforts to **combine different algorithms or integrate them with deep learning** depending on the application.

## S1.1 Deep Learning

Deep learning is a subfield of machine learning that learns abstract representations of data through multi-layered artificial neural networks. **As shown in (S2.a)**, deep learning performs classification directly from raw input without separate preprocessing for feature extraction and selection, whereas machine learning requires distinct stages for feature extraction and feature selection. It demonstrates superior performance especially on large-scale datasets and is particularly effective in handling high-dimensional and unstructured data such as images and speech, often outperforming traditional machine learning approaches. For instance, deep learning-based models have achieved performance at or beyond expert human level in fields such as speech recognition, medical diagnosis, autonomous driving, and cybersecurity. The power of deep learning lies in its hierarchical representation learning, where shallow layers capture low-level features from input data, and deeper layers learn higher-level abstractions, enabling the automatic construction of complex functions.

Representative models of deep learning include **CNNs** and **RNNs**. CNNs are specialized for image

processing and extract local patterns between adjacent pixels using convolutional filters applied hierarchically, making them effective for feature extraction. CNNs have become standard in image classification and object detection, with various architectures such as VGG, ResNet, and DenseNet proposed to improve accuracy (S2.b). On the other hand, RNNs are designed to process sequential data (e.g., time-series signals or text) by introducing a recurrent structure, where outputs from previous steps are fed into the next input to model temporal dependencies. Variants of RNNs include **Long Short-Term Memory (LSTM)** and Gated Recurrent Unit (GRU)networks, which are effective in learning long-term dependencies. CNN- and RNN-based models have significantly advanced applications in computer vision and natural language processing.

According to comparative studies among deep learning models, model performance can vary depending on the data characteristics. For example, in natural language processing tasks, CNN-based models—which excel in recognizing local patterns in sentences—can achieve high performance without recurrence, and it has been reported that CNNs can perform as well as RNNs in short-sentence NLP tasks. Furthermore, another study noted that the superiority of CNN or RNN in NLP tasks depends on the dataset, with CNNs offering faster training due to their ease of parallelization, while RNNs better preserve sequential information. In time-series forecasting, RNN variants such as LSTM have been traditionally used, but in tasks with high volatility, such as stock price prediction, CNNs using sliding windows have shown better accuracy. One study found that CNNs had lower prediction errors than RNNs and LSTMs in predicting the stock prices of three companies, attributing the result to CNN's ability to flexibly respond to changes by focusing on recent information windows rather than past redundant patterns.

In natural language processing, **attention mechanisms** have been introduced in encoder–decoder structures for machine translation, allowing the model to focus on specific parts of the input. The introduction of attention also led to the development of the **Transformer** model, which replaced or supplemented RNNs in some case and resulted in significant performance improvements.

Moreover, new models tailored to specific applications—such as **Temporal Convolutional Networks (TCNs)** and **Kolmogorov–Arnold Networks**—have been proposed. In a comparative study of various deep learning models (CNN, RNN, LSTM, BiLSTM, GRU, TCN, and Transformer), Shiri et al. reported that **Transformers** and **bidirectional recurrent networks** outperformed traditional CNN/RNN models in text analysis. While ResNet and EfficientNet still maintain high performance in image recognition, continuous innovation is underway with architectures such as **Vision Transformers**. Furthermore, multimodal Transformer models such as **CLIP** and **BLIP-2** have been introduced, enabling general-purpose vision–language capabilities through large-scale joint training on text–image data.

In summary, while deep learning can achieve high performance with large-scale data compared to traditional machine learning, its performance is highly sensitive to model architecture and hyperparameters. Therefore, careful design tailored to the problem is necessary. Additionally, deep learning models typically require high computational cost and long training time, making it essential to ensure adequate resources and pay close attention to generalization issues such as overfitting. Nevertheless, deep learning has contributed to solving complex challenges in areas such as image, speech, and language processing, and it continues to dominate much of modern AI research.

(a)

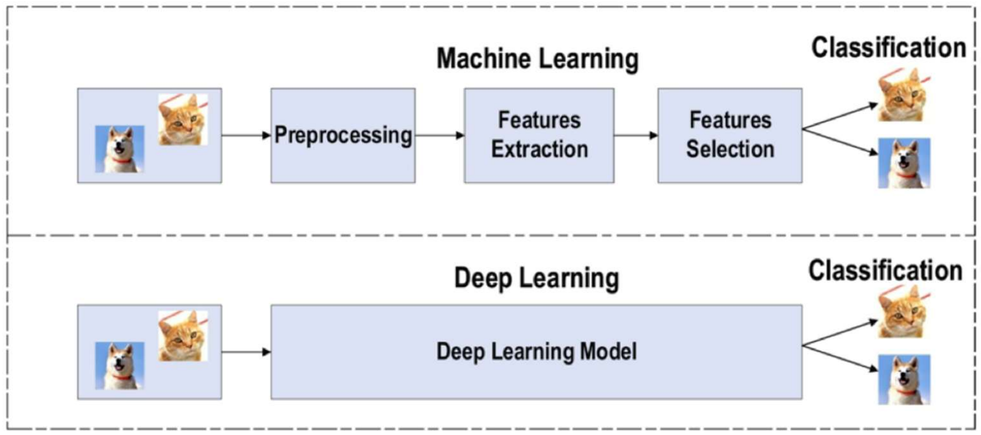


(b)

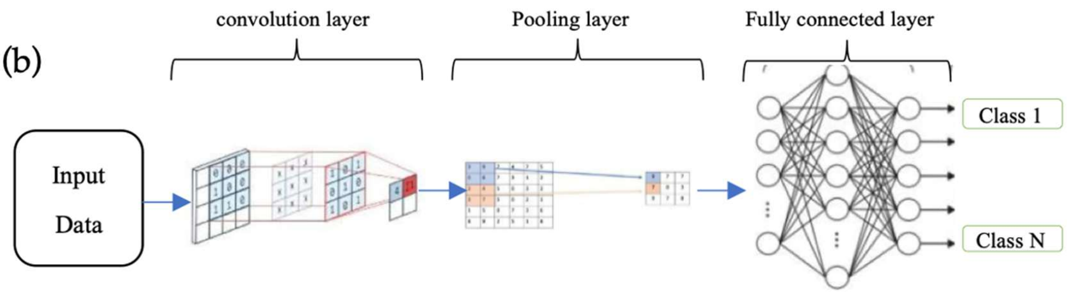


**Figure S 2. (a) Structure of deep learning. (b) Structure of a Convolutional Neural Network (CNN)**

### S1.2. Genetic Algorithms

Genetic Algorithms (GAs) are optimization techniques that mimic evolutionary mechanisms based on natural selection. GAs maintain a population of candidate solutions and evolve them over generations to produce better solutions. In each generation, individuals with higher fitness are selected, new solutions are created through crossover, and diversity is introduced by applying mutation to some individuals. Through repeated evolution, the initially random population is guided toward convergence on the global optimum of the problem.

GAs do not require constraints such as differentiability or continuity, making them applicable to nonlinear and discrete optimization problems. They are particularly effective in exploring complex solution spaces where traditional learning algorithms struggle, and they have also been used for neural network design. For example, **neuroevolution** is an approach that uses GAs to optimize the weights or architectures of artificial neural networks, which is beneficial in scenarios where gradient-based learning methods such as backpropagation are less effective. One study proposed an algorithm that adaptively adjusts mutation probabilities by generation, increasing exploration in the early phase and encouraging convergence in later stages, thus improving efficiency. Moreover, training neural networks using GAs offers advantages such as avoiding local optima and supporting multi-objective optimization. In the work by Tsoy and Spitsyn, a GA capable of optimizing both weights and network structure was developed and successfully applied to logic problems such as XOR.

The most representative neuroevolution algorithm is **NeuroEvolution of Augmenting Topologies(NEAT)**, proposed by Stanley et al. NEAT simultaneously evolves both the structure and weights of a neural network, gradually increasing complexity by adding neurons and synapse

connections across generations. This approach enables the discovery of optimal structures beginning from minimal networks, and it has achieved promising results in various reinforcement learning tasks. An extension of NEAT, **HyperNEAT**, introduces **indirect encoding** to evolve large-scale neural networks more efficiently. In HyperNEAT, a Compositional Pattern Producing Network(CPPN) is evolved to generate the weights of a much larger network as a generative expression. Separately, Buk et al. proposed replacing the topological evolution component of NEAT with **Genetic Programming (GP)** to explore more complex node behaviors. Such evolutionary approaches, including GAs, have been actively applied in neural network design, parameter optimization, feature selection, and beyond. For instance, in a 2024 study, Alfaham et al. applied a NEAT-based evolutionary algorithm to a multiclass classification task, achieving high accuracy by evolving both the architecture and weights of a neural network classifier. Similarly, Mohabeer and Soyjaudah proposed a hybrid method that combines GAs with Radial Basis Function NEAT (**RBF-NEAT)** to improve the efficiency of the evolutionary process. These studies demonstrate that GAs, whether used independently or in combination with other learning techniques, can effectively address complex optimization problems.

Therefore, GAs are considered alternative solutions for problems where traditional algorithms face limitations, due to their advantages in global search capability and parallel exploration. However, they require significant computational resources due to iterative generation updates, and convergence speed can be slow. Additionally, convergence to a global optimum is not guaranteed, so careful parameter tuning (e.g., population size, mutation rate) is essential. Nonetheless, evolutionary approaches—by mimicking biological evolution and adaptation—continue to inspire the design of biomimetic control strategies in robotics, and they are applied in the robot control methods discussed in the following sections.

### S1.3. Reinforcement Learning

Reinforcement Learning is a learning method in which an agent learns a policy to maximize rewards obtained through interaction with its environment. Instead of receiving labeled data, the agent receives reward signals and discovers optimal strategies through **trial and error**, balancing **exploration** and **exploitation**. The **Markov Decision Process (MDP)** framework is commonly used in RL to model decision-making based on cumulative rewards over time.

Traditional reinforcement learning algorithms include value-based methods (e.g., Q-learning), policy gradient methods (e.g., REINFORCE), and actor–critic methods that combine both value and policy learning. In recent years, **DRL** has emerged, combining deep learning with RL to approximate value functions or policies in high-dimensional continuous spaces. A representative example is the **DQN**, which estimates state Q-values using a CNN from image inputs and achieved human-level performance in Atari games. Following this, policy-based deep RL methods have developed, including **TRPO** and its improved version, **PPO**. PPO introduced a **clipping technique** in the actor–critic architecture to improve training stability and performance, and it has become a widely adopted standard in many applications. Meanwhile, **A3C** is an actor–critic method that utilizes multithreaded parallel training to enhance sample efficiency. Although it once led in performance, PPO has since become more dominant in practical use.

According to performance comparison studies of deep RL algorithms, modern algorithms such as PPO often outperform earlier methods like A3C in terms of learning stability and cumulative rewards. Del Río et al. compared A3C and PPO across various general environments and reported that PPO provided better stability and final performance. Similarly, De La Fuente et al. evaluated DQN, PPO, and Advantage Actor-Critic(A2C) in the Atari game *Breakout*, showing performance differences in terms of learning speed, policy development, and adaptability. Specifically, PPO and A2C, being policy-based methods, demonstrated strong performance in continuous control and relatively stable convergence, while DQN, a value-based method, was efficient in discrete action spaces but slower to adapt to complex strategy changes.

In addition, off-policy algorithms for continuous control—such as **SAC** and **TD3**—have been proposed and are gaining attention in robotic control. In a study by Mock et al., PPO, TD3, and SAC were applied to quadruped walking control, and SAC showed the best performance in generating walking patterns. SAC's ability to encourage exploration through entropy-based rewards was considered effective for balancing complex walking motions.

Reinforcement learning has thus evolved into a family of algorithms whose performance depends heavily on the choice of exploration strategies and function approximators. It is critical to select or customize an algorithm that fits the specific nature of the problem. For instance, in robotic control problems with large and continuous state spaces, policy-based methods like PPO or SAC are more suitable than value-based methods like DQN. Conversely, in environments with limited interaction such as game simulations, off-policy algorithms like DQN or Deep Deterministic Policy Gradient(DDPG), which support experience replay, are more appropriate. When input data has temporal dependencies, recurrent structures such as LSTM can be added to the policy network to retain memory of past information. In a study by Tan et al., comparing CNN- and RNN-based deep RL for autonomous navigation, RNN (LSTM)-based agents showed better path-finding performance in environments requiring consideration of temporal changes. As such, the design of RL algorithms and function approximators is closely interconnected and significantly impacts final performance.

Lastly, reinforcement learning enables autonomous agent learning and has been widely applied in various fields such as robotics, game AI, and autonomous driving. The following section explores how different AI algorithms are applied to control problems across various industrial domains, illustrated through concrete case studies.
